# The evolution of lepidopteran brain morphology

**DOI:** 10.1007/s00359-025-01787-w

**Published:** 2025-12-22

**Authors:** Andrea Adden, Susana Garcia Dominguez, Katharina Kliem, Kavitha Kannan, Jothi Kumar Yuvaraj, Tugce Raif, Alejandra Boronat-Garcia, Sara Arganda, Gerard Talavera, Almut Kelber, Stanley Heinze

**Affiliations:** 1 Lund Vision Group, Department of Biology, Lund University, Lund, Sweden; 2 NanoLund, Lund University, Lund, Sweden; 3 Eunice Kennedy Shriver National Institute of Child Health and Human Development, National Institutes of Health (NIH), Bethesda, USA; 4 Universidad Rey Juan Carlos, Departamento de Biología y Geología, Física y Química Inorgánica, Área de Biodiversidad y Conservación, Móstoles, Spain; 5 Universidad Rey Juan Carlos, Instituto de Investigación en Cambio Global (IICG-URJC), Móstoles, Spain; 6 Pheromone Group, Department of Biology, Lund University, Lund, Sweden; 7 Present Address: The Francis Crick Institute, London, UK; 8 Present Address: Institut Botànic de Barcelona (IBB), CSIC-CMCNB, Barcelona, Spain; 9 Present Address: National Institutes of Neurological Disorders and Stroke, National Institutes of Health (NIH), Bethesda, USA

**Keywords:** Evolution, Insects, Neuroanatomy, Immunohistochemistry, Moths, Butterflies

## Abstract

Animals live in diverse environments and have evolved to cope with environmental challenges in different ways. How such adaptations shape overall brain morphology is still unclear. Here, we test how two behavioural adaptations – circadian activity pattern and migratory behaviour – are reflected in the brains of moths and butterflies (Lepidoptera). We predicted that circadian activity pattern affects primary sensory regions, whereas migration impacts integrative centres. Using anti-synapsin immunostaining, we generated detailed 3D reconstructions of each species' brain and performed a phylogenetically corrected volumetric analysis. All lepidopteran brains, including early-diverging lineages, share a characteristic layout that differs from the caddisfly (Trichoptera) outgroup. Some brain regions proved highly evolvable - most notably, the anterior optic tubercle varied qualitatively among species. Most regions, however, differed quantitatively, with tissue volumes strongly shaped by phylogeny as well as behavioural traits. While activity pattern predominantly affected primary visual areas, migratory behaviour correlated with significant volume changes in the fan-shaped body, the accessory medulla and parts of the mushroom body. We also identified several small neuropils as evolutionary “hotspots”, showing rapid, lineage-specific expansion or reduction. Finally, positive and negative correlations among neuropil volumes reveal coordinated evolution in defined neuropil groups, suggesting functional linkages and constraints beyond anatomically related regions. These findings generate testable hypotheses about poorly studied brain areas and highlight diverse evolutionary dynamics across the lepidopteran phylogeny.

**Supplementary Information:**

The online version contains supplementary material available at 10.1007/s00359-025-01787-w.

## Introduction

Brains allow animals to produce behaviour based on sensory information. However, the nervous system is also one of the most energetically expensive tissues. Strong selective pressure therefore exists towards optimally matching the brain's processing power to the sensory and motor capabilities of the animal, producing clear trade-offs between investment in processing power and energetic cost of neural tissue (Niven and Laughlin 2008). This energetic trade-off has been shown particularly for sensory regions, in species as diverse as primates (DeCasien and Higham 2019), fish (Eifert et al. 2015), fruit flies (Keesey et al. 2019) and moths (Stöckl et al. 2016), among others. By comparing closely related species with different sensory behaviours, these studies demonstrated that the primary sensory area of the brain that processes information from the dominant sense was found to be relatively bigger than the same area in a related species, in which this sense is not dominant. For example, diurnal hawkmoths that rely on vision to find flowers have a larger primary visual neuropil than their nocturnal relatives, which rely predominantly on olfaction for the same task (Stöckl et al. 2016).

While this structure-function relationship is relatively direct in primary sensory areas, it has proven more difficult to relate volume changes in integrative brain areas to specific behaviours, particularly in cases where a brain area may underlie several different behaviours (Healy and Rowe 2007). This is especially problematic when it comes to vertebrates, where several studies have related neocortex volume to complex behaviours as diverse as social skills, foraging ecology and migration (review: Healy and Rowe 2007). In insects, specific functions have been attributed to defined integrative areas more reliably (e.g. Menzel 2014; Honkanen et al. 2019; Hulse et al. 2021; Li et al. 2020; Heinze 2024; Donlea 2017), providing a promising starting point for in-depth investigations of the relationship between such brain regions and behaviour.

To date, most work on integrative brain centers was carried out in social hymenopteran insects and almost exclusively focused on the mushroom bodies (MB) (Molina and O’Donnell 2008; Muscedere and Traniello 2012; Amador-Vargas et al. 2015; Ilieş et al. 2015; Valadares et al. 2022; Gowda and Gronenberg 2019). These regions are the insect brain's centres for visual and olfactory learning and memory, and are extremely well-developed in hymenopterans (Menzel 2014). However, as many hymenopteran species are social, the existing data on these integrative neuropils is possibly strongly affected by this ecological parameter, making it crucial to also examine insect orders not impacted by sociality if general insights on principles of brain evolution are to be made. This was recently done in a series of studies on lepidopteran insects (moths and butterflies). While a wide range of species was studied (van Dijk et al. 2017; Snell-Rood et al. 2020; Stöckl et al. 2016), most work examined brain structure across the *Heliconius* genus of butterflies (Montgomery et al. 2016; Couto et al. 2023; Farnworth et al. 2024; Montgomery and Merrill 2017; Farnworth et al. 2025). These studies also focused on the MB or primary sensory neuropils and revealed that advanced navigation capabilities of pollen feeding species were linked to a dramatically increased MB volume (Farnworth et al. 2024; Couto et al. 2023). Significant effects were also found when comparing lab-reared versus wild-caught butterflies within a single species (Montgomery et al. 2016). While highly interesting, the latter results again suggest that the MB is a region that shows unusually high plasticity and might not be representative of the principles guiding brain size evolution in general.

Another study examined a different set of integrative regions of the brain: the central complex (CX), lateral complex (LX) and anterior optic tubercle (AOTU). Here, two species of noctuid moths were compared to illuminate possible effects of migratory behaviour on the neural composition of these regions (de Vries et al. 2017). While this study found no large-scale changes in relative volumes or shape, several smaller differences were reported, suggesting that widening the scope could provide valuable insights into trade-offs shaping sensory and integrative brain regions in insects in the context of complex behavioral traits.

To study how complex behavioral traits are linked to brain structure, the order Lepidoptera proves to be ideal. This order originated as early as 300 million years ago (Kawahara et al. 2019), and comprises 133 families in 43 superfamilies. The Lepidoptera have diversified to give rise to a wide range of sensory-motor capabilities and ecologies. Several species have been studied for decades and have served as models for evolutionary ecology, sensory ecology, motor control and navigation research (e.g. Brakefield et al. 2009; Namiki and Kanzaki 2016; Reppert et al. 2016; Dahake et al. 2018; Beetz et al. 2023; Dreyer et al. 2025; Couto et al. 2023). Additionally, many species are relevant as crop pests.

**Fig. 1:**
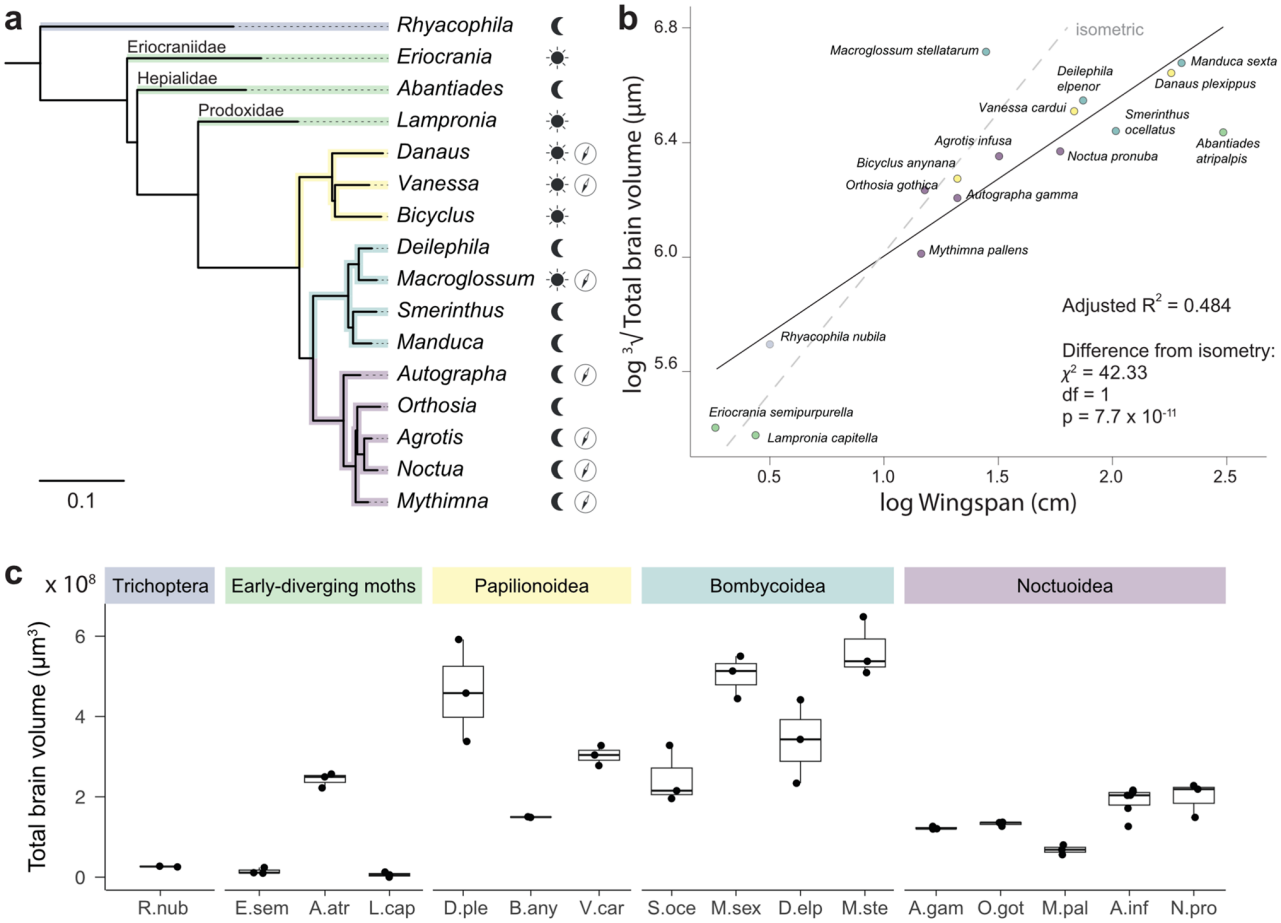
Overview of species included in the study. **a** Phylogenetic tree, including all lepidopteran species used in this study, and the trichopteran outgroup species *Rhyacophila nubila*. **b** PGLS model of total brain volume vs. wingspan. Grey dotted line indicates expected isometric scaling. **c** Total brain volume for all samples and species

We examined 15 different lepidopteran species, covering six families (Fig. 1a). As an outgroup, we additionally included the nocturnal caddisfly *Rhyacophila nubila* (Rhyacophilidae; Zetterstedt 1840), a representative of the Trichoptera, the sister group to Lepidoptera. To maximise the usefulness of our data, we have deliberately chosen species which are used as model systems in other contexts, including pheromone evolution, flight control, or migratory behaviour. Several are economically important pest species or have other exceptional behavioural adaptations, such as a complete loss of adult feeding. Our analysis focuses on two behavioural phenotypes: (1) circadian activity period (diurnal/nocturnal), which allows us to validate our data against previously published results, and (2) migratory behaviour, to understand the potential impact on integrative brain regions. To ensure a balanced dataset, we also included species that show the opposite behavioural phenotype in each family where possible.

By describing the brains of these species, we generated the most diverse collection of lepidopteran insect brains to date (Table 1). We first compared all brain regions across these species on a qualitative level, before performing phylogenetically corrected volumetric analysis. We show that all lepidopteran brains share a similar outline, including those of early-diverging, non-Ditrysian Lepidoptera. Comparing neuropil volumes across species, we found large differences correlating with a diurnal versus nocturnal ecology, but also a set of significant volume changes that were linked to migratory behaviour. Interestingly, a phylogenetically corrected clustering analysis suggests that functionally linked neuropils span anatomically defined superregions, i.e. structurally related neuropil groups (as defined in Ito et al. 2014). Finally, a number of small neuropils emerge as potential hotspots for evolutionary change (Roberts et al. 2022), demonstrating that our analysis has the potential to provide functional hypotheses for hitherto unexplored neuropils.

**Table 1:** Species and ecological traits

Species	Scientific importance	Activity period	Migratory behaviour	Origin	Other
*Rhyacophila nubila* (Rhyacophiloidea, Trichoptera)		Nocturnal	Non-migratory	Wild	
*Eriocrania semipurpurella* (Eriocranioidea)	Larva: pest	Diurnal	Non-migratory	Wild	
*Abantiades atripalpis* (Hepialoidea)	Larva: pulses of biomass	Nocturnal	Non-migratory	Wild	No adult feeding
*Lampronia capitella* (Incurvarioidea)	Larva: pest	Diurnal	Non-migratory	Wild	
*Danaus plexippus* (Papilionoidea)	Adult: long-distance migration	Diurnal	Migratory	Lab	M
*Bicyclus anynana* (Papilionoidea)	Adult: evolutionary genetics	Diurnal	Non-migratory	Lab	M
*Vanessa cardui* (Papilionoidea)	Adult: long-distance migration	Diurnal	Migratory	Lab	M
*Smerinthus ocellatus* (Bombycoidea)	Adult: olfaction, camouflage	Nocturnal	Non-migratory	Lab	No adult feeding
*Manduca sexta* (Bombycoidea)	Larva: pest; Adult: neuroscience, development	Nocturnal	Non-migratory	Lab	Hovering flight
*Macroglossum stellatarum* (Bombycoidea)	Adult: vision, flight control	Diurnal	Migratory	Lab*	Hovering flight
*Deilephila elpenor* (Bombycoidea)	Adult: vision	Nocturnal	Non-migratory	Lab*	Hovering flight
*Autographa gamma* (Noctuoidea)	Larva: pest	Nocturnal	Migratory	Wild	
*Agrotis infusa* (Noctuoidea)	Larva: pest; Adult: long-distance migration	Nocturnal	Migratory	Wild	
*Noctua pronuba* (Noctuoidea)	Larva: pest	Nocturnal	Migratory	Wild	
*Mythimna pallens* (Noctuoidea)		Nocturnal	Migratory	Wild	
*Orthosia gothica* (Noctuoidea)		Nocturnal	Non-migratory	Wild	

*M* male-released pheromone. If pheromone is not specified, it is female-released. Origin indicates whether a species was wild-caught or lab-reared, where lab* denotes that the captive population was regularly replenished with wild-caught individuals and larvae were reared on their natural food plants

## Material and methods

### Animals

All sampled animals were male, with the exception of *V. cardui* where sex was unknown. We sampled two or three brains per species for all species except *A. infusa*, of which we included six samples. Note that species availability required us to use a mix of lab-reared and wild-caught animals throughout the study (Table 1). While this difference in origin has the potential to impact neuropil volumes, we were unable to include it as a variable in our analysis, because origin correlated with phylogenetic group in our dataset (Table 1). However, as inter-specific variation (impact of phylogeny) is expected to out-size the effect of origin (Montgomery et al. 2016; Couto et al. 2023) and our analyses focus on inter-species comparisons, we expect no major effects on any of our conclusions.

#### Noctuoidea

Silver Y moths (Noctuidae, Plusiinae: *Autographa gamma* Linnaeus 1758), yellow underwings (Noctuidae, Noctuini: *Noctua pronuba* Linnaeus 1758), common wainscots (Noctuidae, Hadeninae: *Mythimna pallens* Linnaeus 1758) and hebrew characters (Noctuidae, Hadeninae: *Orthosia gothica*) were caught in lepiLED light traps (Brehm, 2017) near Lund (Sweden). No collection permits were needed. Bogong moths (Noctuidae, Noctuini: *Agrotis infusa* Boisduval 1832) were collected during their aestivation period in caves located in Kosciuzko National Park (New South Wales, Australia) under a Scientific Licence issued by the New South Wales National Parks and Wildlife Service (no. SL100806).

#### Bombicoidea (Hawkmoths)

Elephant hawkmoths (Sphingidae, Macroglossinae: *Deilephila elpenor* Linnaeus 1758) were purchased as pupae from a commercial supplier (Neil West, Newark, UK) and stimulated to eclose by transferring them from 4$^{\circ }$ C to room temperature. Hummingbird hawkmoths (Sphingidae, Macroglossinae: *Macroglossum stellatarum* Linnaeus 1758) were taken from a lab-reared population, based on wild-caught individuals collected in Sorede (France). *D. elpenor* and *M. stellatarum* adults were fed 10% sugar solution and allowed to fly at least once after eclosion before they were collected for dissection. The brains obtained from these species were previously analysed in Stöckl et al. (2016).

Eyed hawkmoths (Sphingidae, Smerinthinae: *Smerinthus ocellata* Linnaeus 1758) were purchased as larvae from a commercial supplier (Worldwide Butterflies, Lulworth, UK). The larvae were raised on apple leaves. Adults were allowed to fly at least once after eclosion before they were collected for dissection.

Tobacco hornworms (Sphingidae, Sphinginae: *Manduca sexta* Linnaeus 1763) were reared from eggs (Carolina Biological Supply Company, USA) on an artificial diet at 26$^{\circ }$ C in 70% humidity, under a long-day photoperiod. For eclosion, individual pupae were placed in plastic cups containing a mesh that allows newly eclosed moths to extend their wings. Only moths with fully extended wings, intact proboscis and antennae, and an overall healthy appearance were used for dissection.

#### Papilionoidea (Butterflies)

Monarch butterflies (Nymphalidae, Danainae: *Danaus plexippus* Linnaeus 1758) were ordered as pupae from Costa Rica Entomological Supply and kept in an incubator (HPP 110 Memmert GmbH + Co. KG, Schwabach, Germany) at the University of Würzburg (Germany) at 25$^{\circ }$ C, 80% relative humidity and a 12:12 h light/dark cycle. After eclosion, adult Monarch butterflies were transferred into another incubator (I-30VL, Percival Scientific, Perry, IA, USA) at 25$^{\circ }$ C and 12:12 light/dark conditions, where they had access to 15% sucrose solution *ad libitum*. Squinting bush browns (Nymphalidae, Satyrinae: *Bicyclus anynana* Butler 1879) were derived from a lab-bred population maintained at the University of Cambridge (courtesy of Dr. Oskar Brattström). Painted ladies (Nymphalidae, Nymphalinae: *Vanessa cardui* Linnaeus 1758) were obtained from a 3rd-4th generation laboratory bred line, that was derived from a field-collected population (origin North America). These were raised in growing chambers with thistles as a hostplant. Animals were either bred under long day, warm conditions (n = 2, 14 h daylight, 30$^{\circ }$ C day, 20$^{\circ }$ C night) or under short day, cold conditions (n = 1, 8 h daylight, 20$^{\circ }$ C day, 12$^{\circ }$ C night). Dissections were performed 2-3 days after eclosion.

#### Early-diverging Lepidoptera and Trichoptera

Currant shoot borer moths (Incurvarioidea, Prodoxidae: *Lampronia capitella* Clerck 1759) were collected from a blackcurrant plantation near Roskilde (Denmark). Birch leafminer moths (Eriocranioidea, Eriocraniidae: *Eriocrania semipurpurella*) were caught in live pheromone traps in a Birch forest 15 km East of Lund (Sweden). As this method predominantly traps male moths, this study was restricted to males for all species. Rainmoths (Hepialoidea, Hepialidae: *Abantiades atripalpis*) were caught shortly after emergence in light traps near Adaminaby (New South Wales, Australia). Pupae of the caddisfly *Rhyacophila nubila* (Trichoptera, Rhyacophilidae, Zetterstedt 1840) were collected from a stream near Sjöbo (Sweden) and kept in aerated aquaria at 14-16$^{\circ }$ C and a 16:8 h light:dark cycle until the adults emerged. No collection permits were needed for these species.

### Wingspan measurements

The wingspan was measured directly by adding the length of both wings to the width of the thorax between the two wing joints, for *A. gamma*, *N. pronuba*, *S. ocellatus* and *M. pallens*. This could not be done for the other species, as we re-used samples for which the bodies were not preserved. Instead, we obtained an approximation of the wingspan from published papers, moth identification books and public websites, specifically (Skinner and Wilson 2009) for *D. elpenor* and *M. stellatarum*, (Warrant et al. 2016) for *A. infusa*, (Novak and Severa 1980) for *E. semipurpurella*, https://www.ukmoths.org.uk/species/lampronia-capitella/ for *L. capitella*, https://www.ukmoths.org.uk/species/orthosia-gothica/adult/ for *O. gothica*, (Braby 2000) for *D. plexippus*, (Brakefield et al. 2009) for *B. anynana*, (Arnett and Jacques 1981) for *M. sexta*, and https://www.lepidoptera.se/species/vanessa_cardui.aspx for *V. cardui*. Wingspan was the only size measure available for all species, yet we note that it is only an approximation of body size, given wingspan is also impacted by varying flight styles.

### Histology

#### Antibodies

In the following histology protocols, we used an anti-synapsin antibody which was kindly provided by Dr. E. Buchner (Würzburg University, Germany; Cat# SYNORF1 (*Drosophila* synapsin I isoform), RRID: AB_2315426; Klagges et al. (1996)). The secondary antibody was GAM-Cy5 (goat anti mouse conjugated to Cy5; Jackson Immunoresearch Laboratories Inc., West Grove, PA, USA; Cat# 115-175-146, Lot# 108262), and Alexa-568 (goat anti mouse conjugated to Alexa 568; ThermoFisher, A11031) for *M. sexta*. Normal goat serum (NGS; Jackson Immunoresearch, West Grove, PA, USA; Cat# 005-000-121) was used for blocking non-specific antibody binding sites.

#### Immunohistochemistry protocol

The whole-mount staining protocol was adapted from the protocols described in Heinze and Reppert (2012), Ott (2008) and Stöckl and Heinze (2015). Cold anesthetised animals were killed by severing the head from the thorax. Noctuid, hepialid, hawkmoth and butterfly heads were mounted in a wax-filled petri dish and dissected to expose the brain. Fresh ZnFA fixative (18.4 mM ZnCl$_2$, 135 mM NaCl, 35 mM sucrose, 1% paraformaldehyde; Ott (2008)) was immediately applied to the brain, which was then dissected out of the head capsule, cleaned of trachea and fat, and the retina was removed. The brains of the smaller species were dissected from freely floating heads in ZnFA fixative.

Brains were left to fix for 20 h at 4$^{\circ }$ C (room temperature for *V. cardui*) and were then washed 6-8x20 min in HEPES-buffered saline (HBS: 150 mM NaCl, 5 mM KCl, 5 mM CaCl$_2$, 25 mM sucrose, 10 mM HEPES; Ott (2008)). In the case of *D. elpenor*, *M. stellatarum* and *A. infusa*, brains were bleached in 10% H$_2$O$_2$ in Tris-buffered saline (Tris–HCl) for 8 h (Stöckl and Heinze 2015). However, this step was skipped for all other species. Brains were subsequently washed in Tris–HCl (3x10 min; once for *V. cardui*), permeabilised in a fresh mixture of dimethyl sulfoxide (DMSO) and methanol (20:80) and washed again in Tris–HCl (3x10 min; omitted for *V. cardui*). This permeabilization step was adjusted in duration to different sized brains (from 30 to 75 min).

After pre-incubating the brains in 5% normal goat serum (NGS) in 0.01 M phosphate-buffered saline (PBS) with 0.3% TritonX-100 (PBS-Tx) over night at 4$^{\circ }$ C (1-2 h at room temperature for *V. cardui*), brains were transferred to the primary antibody solution (anti-synapsin 1:25, 1% NGS in PBS-Tx; 1:30 for *V. cardui*), and left to incubate at 4$^{\circ }$ C (room temperature for *V. cardui*) for 3-6 days (depending on species). Brains were washed 8x20 min in PBS-Tx (6x10 min for *V. cardui*) before being transferred to the secondary antibody solution (1:300 GAM-Cy5, 1:250 GAM-AlexaFluor-568 (*M. sexta*), 1:100 GAM-AlexaFluor-488 (*V. cardui*)), 1% NGS in PBS-Tx), and left to incubate at 4$^{\circ }$ C for 2-5 days (room temperature for *V. cardui*).

Brains were again washed in PBS-Tx (6x15 min) and PBS (2x15 min) and dehydrated in an increasing ethanol series (50%, 70%, 90%, 96%, 2x100%, 15 min each) and, for *M. sexta*, in glycerol series (8%, 15%, 30%, 50%, 60%, 70%, and 80%, 2–24 h) diluted in Tris buffer with 1% DMSO. For clearing, brains were transferred to a fresh mixture of ethanol and methylsalicylate (1:1) and the ethanol was allowed to evaporate. After 15 min, the mixture was replaced by pure methylsalicylate, in which the brains were left to clear for 30 to 75 min (depending on species). After clearing, the sheath covering the brains of *M. sexta* brains was carefully removed with forceps. Finally, the brains were embedded in Permount (Electron Microscopy Science, Hartfield, PA, USA) between two coverslips, using plastic spacers (Zweckform No. 3510, Germany) to prevent squeezing. For *V. cardui* brains were mounted directly in methylsalicylate, with metal washers serving as spacers.

#### Confocal imaging

Images were taken with a confocal laser scanning microscope (Leica SP8 DLS, Leica Microsystems GmbH, Wetzlar, Germany, or Zeiss LSM 510 Meta, Carl Zeiss AG, Oberkochen, Germany). For 3D reconstructions, whole-mount brains were imaged using the 633nm HeNe laser and a 25x objective (LD LCI Plan-Apochromat 25x/0.8 DIC Imm Corr, Zeiss) or a 20x objective (HC PL APO CS2 20x/0.75 IMM, Leica). *V. cardui* image stacks were acquired separately, using a Nikon C2 confocal microscope (Nikon, Melville, NY) with a 10x objective (numerical aperture 0.45). The z-compression resulting from refractive index mismatches between immersion and mounting media was corrected digitally by multiplying the z-dimension of the image stacks with the ratio of the refractive indices (most relevant for 10x air objective used for *V. cardui)*. Scans of whole-mount preparations were acquired as stacks of 1 $\mu$m thick optical sections at 1024x1024 resolution. In the x-y dimension, voxel sizes were variable between species. They were set to values below the ultimately required voxel size for 3D reconstructions (between 0.5 and 2 $\mu$m), yet as large as possible to maximise photon capture and minimise scan time. Detector gain and offset, as well as laser power were adjusted for each sample in order to optimally expose the image. Scans were aligned and merged either using the Nikon microscope software (*V. cardui*) or, for all other species, using the stitching tool in the ImageJ implementation FIJI (general public license, downloadable from fiji.sc), using the stitching algorithm based on Preibisch et al. (2009). Scans from anterior and posterior were later aligned by affine registration and merged in the 3D reconstruction software Amira 5.3.3 (FEI, Hillsboro, OR, USA).

### Three-dimensional reconstructions

The neuropil reconstructions shown in this paper were done in the 3D reconstruction software Amira 5.3.3. Each image stack was downsampled to voxel sizes between 0.5x0.5x1 $\mu$m and 2x2x2 $\mu$m (smaller for smaller species). Using the segmentation editor of Amira, we then manually segmented individual cross sections of each neuropil in all three spatial planes. The full structure of reconstructed neuropils was interpolated using the “wrap” function. For approximately half the brains in the dataset an updated workflow was used. Here, we manually labeled cross sections of all neuropils only in the imaging plane, using identical planes for all neuropils. Together with the image stack, the resulting Amira label field file was uploaded to the online tool Biomedisa (Lösel et al. 2020), where the full 3D structure of each neuropil was computed by using the image data to expand the manually segmented seed layers. The results were smoothened, re-imported into Amira and any segmentation errors were manually corrected. This updated workflow was not only orders of magnitude faster, but also yielded more realistic neuropil boundaries.

For both workflows we extracted neuropil volumes using the “MaterialStatistics” function in Amira. We finally generated polygonal surface models to visualise the neuropils in Amira. Direct volume renderings of neuropils were carried out in 3D-slicer (https://www.slicer.org/; (Fedorov et al. 2012)) after masking image stacks using neuropil segmentation data. Type samples of each species can be accessed on http://www.insectbraindb.org.

Neuropil identification was done by comparing location and shape of brain regions to described lepidopteran insects as well as other insects. The lobes of the MB were identified by comparing specifically to the data provided in Sjöholm et al. (2005). As we were not able to disentangle the $\alpha$ from $\alpha '$ lobes and the $\beta$ from $\beta '$ lobes, those were treated as units (vertical and medial lobes). The $\gamma$-lobe was assigned to the most anteriorly located lobe section and split into vertical and medial parts. Especially in the more basal species, the assignment of lobes remains preliminary and confirmation by detailed characterization of Kenyon cell morphology would be required to eliminate remaining uncertainties. In particular, the lack of identified lobes in some species might not necessarily mean that the corresponding Kenyon cells do not exist, but merely that the region did not stand out at the neuropil level.

Naming of neuropils follows the conventions defined in Ito et al. (2014).

### Data analysis

All volumetric data was analysed in RStudio (version 2024.12.0+467). Paired neuropils were summed to obtain a single total volume per neuropil for each individual brain. The values presented in this paper are the mean and standard deviation of all brains for that species. Unless specified otherwise, the values shown are the relative values of neuropils, normalised to the total volume of the central brain, which was obtained by summing the undefined neuropils (superior, ventrolateral, ventromedial, inferior, circumesophageal, and gnathal neuropils). We used the undefined neuropils of the central brain as reference volume for normalization, as this ensures that no dependent variables are included in the allometric control.

The phylogenetic tree shown in figure 1a and used in our analysis was redrawn from Kawahara et al. (2019). Species not included in Kawahara et al. (2019) were added by replacing their closest phylogenetic relation as follows: *Noctua pronuba* replaces *Striacosta albicosta* (tribe Noctuini); *Mythimna pallens* replaces *Mythimna separata* (genus Mythimna); *Macroglossum stellatarum* replaces *Macroglossum pyrrhosticta* (genus Macroglossum); *Smerinthus ocellatus* replaces *Smerinthus saliceti* (genus Smerinthus); *Vanessa cardui* replaces *Polygonia c-album* (tribe Nymphalini); *Lampronia capitella* replaces *Tegeticula yuccasella* (family Prodoxidae); *Abantiades atripalpis* replaces *Cibyra sp.* (family Hepialidae); *Eriocrania semipurpurella* replaces *Dyseriocrania subpurpurella* (family Eriocraniidae); *Rhyacophila nubila* replaces *Palaeagapetus nearcticus* (suborder Integripalpia); *Autographa gamma* replaces *Amphipyra pyramidea* (based on Li et al. 2024); *Orthosia gothica* replaces *Sesamia inferens* (based on Keegan et al. 2022); and *Deilephila elpenor* replaces *Hyles euphorbiae* (based on Wright et al. 2024).

To estimate phylogenetic signal, we chose Blomberg’s *K* as it is least sensitive to small sample sizes (n=15 in our analysis) (Münkemüller et al. 2012). *K* was calculated for each neuropil individually using the phytools package in R (Revell 2023). To test the hypothesis $K = 0$, we used the *phylosig* function’s random permutation test with 10,000 simulations. To test the hypothesis $K = 1$, we simulated the distribution of *K* under a Brownian Motion model of evolution (10,000 simulations) and performed a two-sided test of the observed *K* against the simulated *K*.

For analysing the allometric relationship between species and neuropils while accounting for phylogenetic distance, we performed phylogenetic generalised least squares (PGLS) analysis using the phylolm (10.32614/CRAN.package.phylolm) and phytools (Revell 2023) packages. We assumed the standard allometric relationship $y = a x^b$, which can be expressed as the linear relationship

$$log(y) = b \times log(x) + log(a).$$

If the neuropils scaled isometrically across species, the regression slope b would be expected to equal 1. We express this as the slope index

$$\begin{aligned} si = b - 1 \end{aligned}$$

where si >0 indicates a steeper than isometric scaling. To analyse how ecological parameters are reflected in the relative volumes of individual brain areas, we calculated the grade shift between the regressions for species subgroups that differ in an ecological trait. Thus, we tested for differences in the y-axis intercept log(a) given no significant difference in the slope b. Intercept differences are given as the grade shift index

$$\begin{aligned} gsi = \frac{a_{ecology1}}{a_{ecology2}} - 1 \end{aligned}$$

where gsi >0 indicates that neuropil volumes are systematically larger in the ecology 1 subgroup. To test for significant differences in si and gsi between ecologies, we used the car::linearHypothesis Chi-squared test function in R. Note that PGLS assumes that inter-specific variation is greater than intra-specific variation, which we confirmed by comparing the variation of neuropil volume within *A. infusa* across six samples to the variation across species.

## Results

To reveal differences and similarities between the brains of the 16 different species included in this study (Fig. 1a, S1), we generated 3D reconstructions based on confocal image stacks of anti-synapsin immunolabeled brains. Reconstructions were based on three brains per species, with the exception of *B. anynana* and *R. nubila*, where only two samples were available. Image segmentation was carried out using identical criteria for all samples in the dataset. The reconstructed neuropils included all clearly defined brain areas with all identifiable subdivisions, identical to the average atlas of the Bogong moth brain (Adden et al. 2020). As the lamina of the optic lobe could not be preserved in all individuals, it was excluded from our analyses. We also refrained from dividing the large mass of undefined neuropil of the central brain into its components, as its internal borders are ambiguous and clear homologies between species are difficult to infer, making quantitative comparisons of neuropil volumes problematic.

### Brain layout across species

First, we examined the total size of all brains. Across all species, the brains covered a wide range of sizes, from a volume of approximately 9,300,000 $\mu m^3$ in *E. semipurpurella* to approximately 600,000,000 $\mu m^3$ in *M. sexta* (Fig. 1c). The largest brain is thus almost 65 times larger than that of the smallest species. Across that range, the absolute brain volume increased with body size, measured as the wingspan (PGLS regression: $R^2$ = 0.484; Fig. 1b). The slope of this regression was significantly shallower than would be expected for isometric scaling ($\chi ^2$ = 42.33, df = 1, p < 0.001), suggesting that there is diminishing benefit from increasing brain size in increasingly large lepidopteran species. While the majority of the examined species followed the regression well, including the trichopteran species used as outgroup, the hawkmoth *M. stellatarum* possessed a brain nearly twice the volume of similarly sized other species in the dataset, while the rainmoth *A. atripalpis*’ brain was only half as big as expected by body size. The latter is likely due to the decreased behavioural complexity and short lifespan of the adults, which lack mouth parts and do not feed. Indeed, the same effect can be seen in the eyed hawk moth *S. ocellatus*, which has a similarly reduced behavioural repertoire. Interestingly, *M. stellatarum* is the only species in our dataset that combines migratory ability with a diurnal activity period, within an otherwise nocturnal phylogenetic group, suggesting that additional neural requirements might emerge from this trait combination. Diurnal activity and migratory ability also coincide in two of our butterfly species (*D. plexippus* and *V. cardui*), however a direct comparison to hawkmoths is complicated by the different flight styles of butterflies and hawkmoths, yielding different relationships between body mass and wing span.

**Fig. 2:**
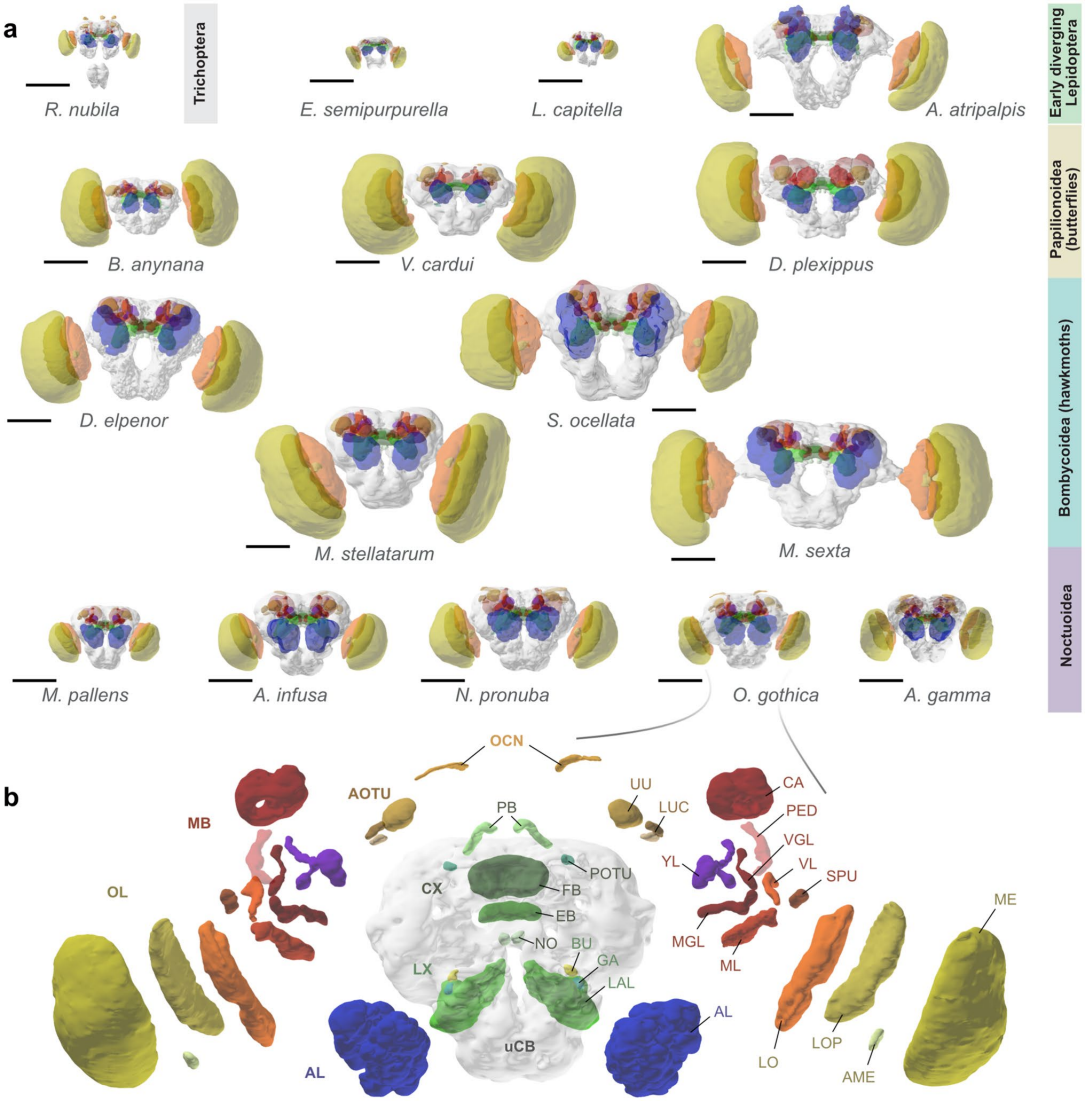
The brains of all species included in this study. **a** 3D reconstructions based on anti synapsin immunolabeling. Shown is an individual example of each species, except for the Bogong moth (*Agrotis infusa*), which is represented by an average shape brain (from Adden et al. (2020)). The lamina of the optic lobe was only intact in a small number of samples and was thus excluded from the analysis. **b** All neuropils included in the 3D reconstruction, shown individually, using the brain of *Orthosia gothica* as example. Abbreviations on the left indicate super-regions, while abbreviations on the right show neuropils. Scalebars in a: 500 $\mu$m; All images were generated using http://www.insectbraindb.org (Heinze et al. 2021)

**Fig. 3:**
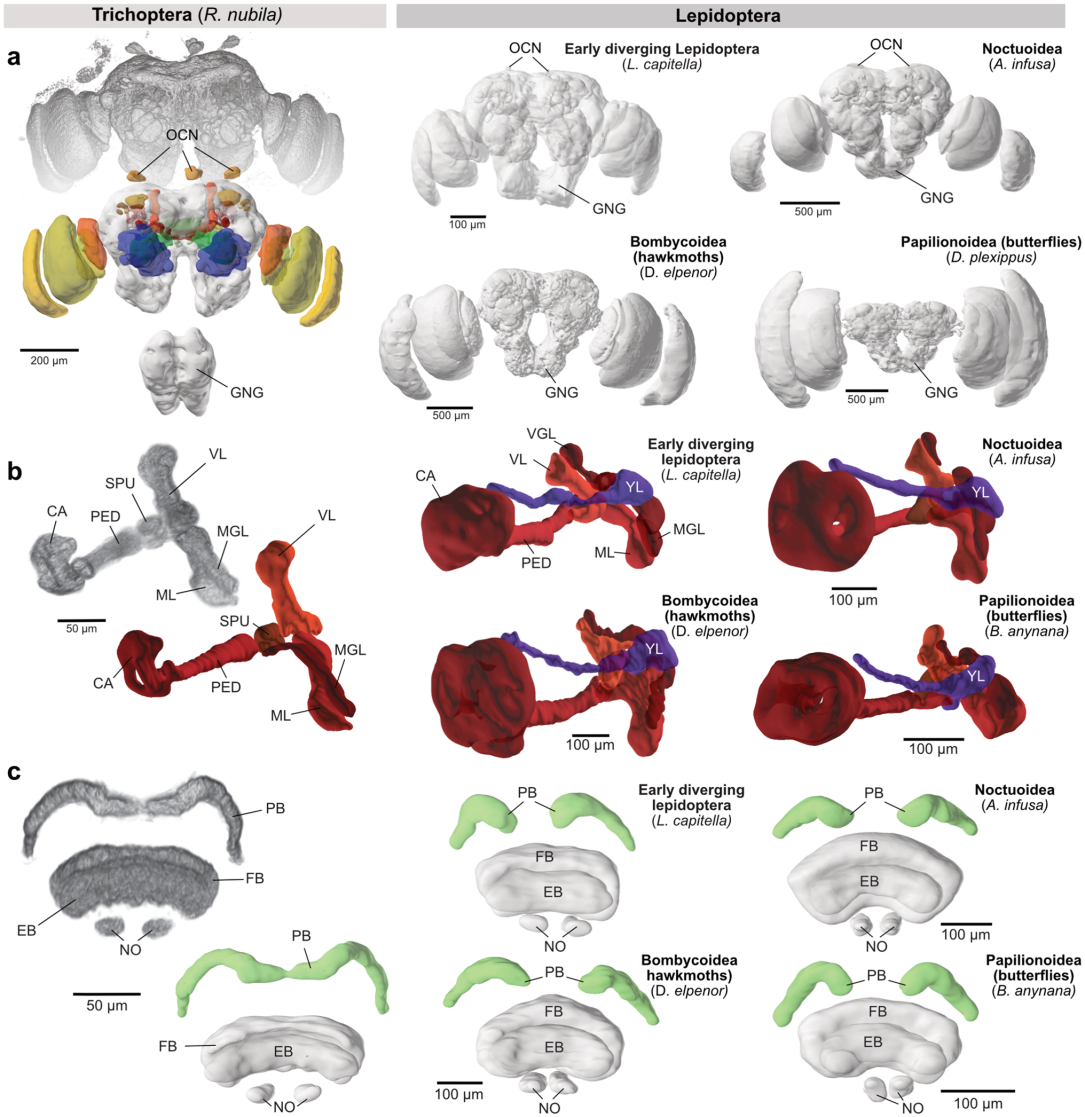
Defining features of lepidopteran brains. **a** Left: Morphology of a Trichopteran brain, illustrated by volume rendering and 3D reconstruction of the brain of *R. nubila*, based on anti synapsin immunolabeling. Right: Brain reconstructions of species representing the four families of Lepidoptera examined in this study. As opposed to Trichoptera, all lepidopteran brains share a gnathal ganglion (GNG) that is fused to the main brain (cerebral ganglion), as well as reduced ocellar neuropils. Instead of three ocellar neuropils (OCN), Lepidoptera either possess two small (sometimes vestigial) or no ocellar neuropils. **b** The lepidopteran mushroom body features an additional lobe system, the Y-lobes, composed of an accessory pedunculus (Y-tract) and the dorsal and ventral lobelets, together shown in purple. All remaining mushroom body components are similar across both insect orders, with the vertical $\gamma$-lobe (VGL) not individually recognizable in *R. nubila*, but also in several lepidopteran species. **c** The central complex is highly similar across both insect orders, with the exception of the protocerebral bridge (PB), which is continuous across the midline in Trichoptera, but split into two hemispheres in lepidoptera. Images of 3D reconstructions generated with http://www.insectbraindb.org. For abbreviations, see the Abbreviations section

We next investigated the general arrangement of brain neuropils across our species. All lepidopteran brains were very similar in their overall shape, as well as in the location and relative orientation of neuropils within the brain (Fig. 2), indicating a commonly shared lepidopteran ground plan. To test this idea, we examined the brain of *R. nubila*, a representative of the Trichoptera, the sister group of the Lepidoptera. This species indeed showed several features that were not shared by any of the lepidopteran brains: The gnathal ganglion was not fused with the cerebral ganglion, three ocelli and their corresponding ocellar neuropils were present, the protocerebral bridge of the CX was fused across the midline, and a secondary pedunculus of the mushroom body (Y-tract/lobe) was absent (Fig. 3, S3).

Generally, these trichopteran traits are typical for many other insects and indicate that several lepidopteran brain features are commonly derived characteristics of this insect order. These include a split protocerebral bridge, the existence of a secondary pedunculus (Y-lobe) of the mushroom body, and the loss of the median ocellus.

Given the large qualitative differences between lepidopteran brains compared to their trichopteran sister group, we restricted all detailed quantitative analyses to our 15 lepidopteran species.

### Qualitative differences across lepidopteran brains

**Fig. 4:**
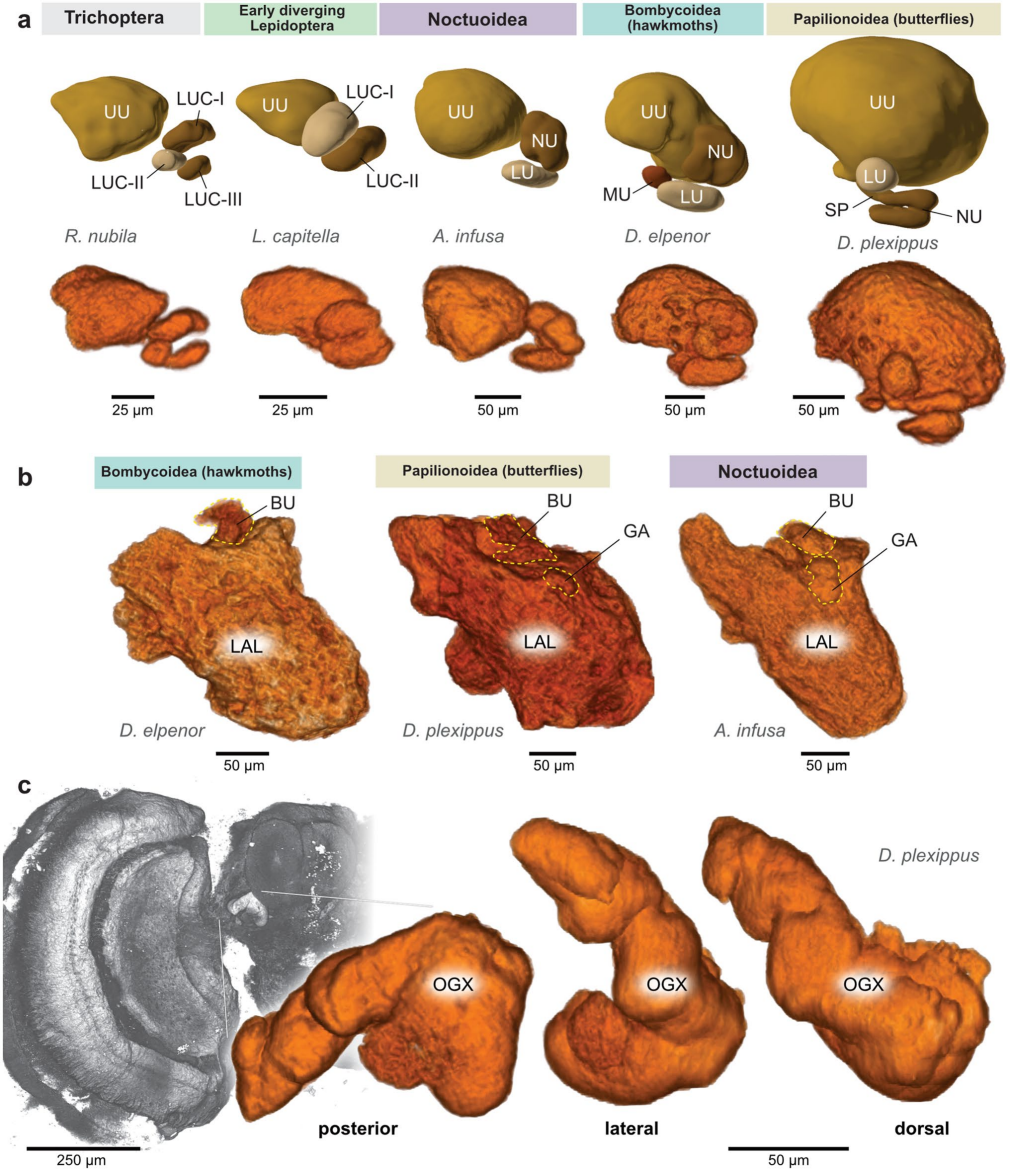
Qualitative differences in neuropil composition. **a** The composition of the lower unit complex (LUC) of the anterior optic tubercle (AOTU) varies significantly between major phylogenetic groups. The top row shows 3D reconstructions based on anti synapsin immunolabeling, while the bottom row directly depicts volume renderings of the underlying image data. Images of 3D surface reconstructions generated with http://www.insectbraindb.org. **b** Volume rendering of the lateral complex (LX) of species representing three major groups of Lepidoptera. The gall (GA) is comparably large and complex in Noctuoidea, but non-existent in Bombycoidea (hawkmoths). In Papilionoidea (butterflies) it is distinct, but small. **c** The optic glomerular complex (OGX) is a neuropil only found in the Monarch butterfly. Location and shape is illustrated by volume renderings of image data

Despite having highly conserved neuropil architectures across the four major groups of Lepidoptera, our data revealed a limited set of qualitative differences (Fig. 4). Most prominently, the lower unit complex of the anterior optic tubercle (AOTU) showed a varying subunit composition that correlated with the phylogenetic group that each species belonged to. In noctuid moths, we consistently found the well-described arrangement of one nodular unit (consisting of four individual nodules) and one lower unit. However, this composition was slightly different in all other groups. In hawkmoths, we identified a previously undescribed sub-compartment, here termed the medial unit, located at the medial edge of the lower unit complex, close to the upper unit (Fig. 4a). In butterflies the overall shape of subunits diverged substantially from that found in moths. Our nomenclature followed that used by Heinze and Reppert (2012), in which homologies were inferred by location and innervation patterns of tubercle-bulb neurons (Heinze et al. 2013). The small additional subunit identified in the Monarch butterfly, the strap, was confirmed in our data, but was not found in the other two butterfly species used, demonstrating variability in the layout of this neuropil within butterflies.

In early-diverging Lepidoptera and Trichoptera, two to three subdivisions of the lower unit complex were found. However, due to the large variations in shape, location and size compared to those found in the other lepidopteran groups and the lack of neuronal innervation data, we did not infer speculative homologies. All quantitative analyses thus treated the lower unit complex as one single entity. Yet, this region, which serves as a main processing stage for navigation-relevant visual information, is clearly evolutionarily more variable than most other neuropils.

A second anatomical feature correlating with phylogenetic group is the presence and shape of the gall, a small compartment of the lateral complex, receiving axons from EPG head direction cells. This region was consistently absent in hawkmoths (Fig. 4b, S4). However, whether it does not exist or is merely fused with the lateral accessory lobes is an open question that requires data from single cell morphologies. In contrast to hawkmoths, in noctuid moths the gall is relatively large and can be divided into distinct dorsal and ventral compartments (Fig. 4b). This arrangement resembles that in flies and beetles, in which EPG neurons from even and odd columns of the protocerebral bridge target either the dorsal or the ventral gall compartment (el Jundi et al. 2018; Hulse et al. 2021).

The other lepidopteran groups showed gall morphologies in between the two extremes. In butterflies, a single small, yet distinct, region was found, which in the Monarch butterfly has been described to possess a unique glomerular structure (also called the anterior lobelet of the lateral accessory lobes; Heinze and Reppert (2012)). In early-diverging Lepidoptera and Trichoptera, the gall was visible, but did not show a division into dorsal and ventral gall, indicating a simpler overall structure.

Two more regions were variable across our species: The ocellar neuropils and the posterior optic tubercle. Ocellar neuropils were largest in noctuid moths (Fig. 2b). In butterflies and hawkmoths, the ocellar neuropils were not recognisable at the resolution used for neuropil reconstructions, and thus might either not exist or have been reduced to vestigial size, corresponding to very small or internalised ocelli in those insects (Dickens and Eaton 1973; Eaton 1971). Early-diverging moths frequently showed asymmetries, with the ocellar neuropil on one side of the brain being much larger than on the other side. However, no consistent side bias was found across individuals or species.

The posterior optic tubercle was present in most species, including *R. nubila* (Fig. S4). Exceptions were found within three groups: Hawkmoths, early-diverging species and butterflies. However, we were not able to resolve this region in the Monarch butterfly, even though it has been confirmed to be present in previous work (Heinze and Reppert 2012; Heinze et al. 2013). This demonstrates that for very small, irregular shaped neuropils our image resolution was not always sufficient to reliably identify these structures. Similarly, in all early-diverging Lepidoptera, the posterior optic tubercle was inconsistently found across individuals of the same species, suggesting either that this neuropil is fused with surrounding regions (posterior protocerebrum or protocerebral bridge) to varying degrees, or different amounts of background staining at the edge of the brain has hindered consistent detection. In contrast, in hawkmoths, the posterior optic tubercle was consistently found in *M. stellatarum* and *S. ocellatus*, but consistently not found in *M. sexta* and *D. elpenor*, indicating repeated loss of this region within the Bombycoidea.

Finally, one large neuropil was only found in the Monarch butterfly: the optic glomerular complex (Fig. 4c). This region has been described earlier (Heinze and Reppert 2012) and comprises a dense patch of segregated neuropil stretching from the inner rim of the lobula towards the lateral edge of the lateral calyx of the mushroom body. While it was prominent in all individuals of the Monarch butterfly, no comparable region was present in *B. anynana* and only a much smaller neuropil near the lobula was found in *V. cardui*, which did not stretch into the optic stalk. This region was complemented by a synapsin-rich, yet irregular area in the ventrolateral protocerebrum. Given the limited resolution of the dataset, these neuropils were not segmented.

### Phylogenetic distance is an important factor determining brain differences

While we found some qualitative changes across our species, most neuropils varied in size, i.e. differences between species were quantitative in nature. It is tempting to interpret such size differences as adaptations to the computational requirements imposed by differences in ecological traits. However, environmental pressures act on species that share a phylogenetic history. Brains of closely related species may therefore be more similar than brains of distantly related species, sharing a more recent common layout and allowing for shorter time frames in which brains evolved independently. While this does not preclude rapid evolutionary change, phylogenetic distance is clearly a factor that needs to be accounted for.

For all quantitative analyses, each neuropil of each species is represented by a single data point, indicating the mean volume of this region. To show that our inter-species comparisons are meaningful and are not impacted by inter-individual variability, we additionally compared the variability of all brain regions within species to their variability across species (Fig. S5). We found that levels of within-species variability are generally comparable for all species and that inter-species variability exceeds intra-species variability by at least one order of magnitude (Fig. S5d, e). Hence, even with low sample numbers per species, between-species effects can be expected to dominate the results, and should not be significantly affected by variability between individuals of the same species. Additionally, small neuropil volumes are generally more prone to reconstruction error, as small voxel misallocations affect the overall neuropil volume more strongly in regions with fewer overall voxel numbers. However, this effect on increased variability for small neuropils was only visible within species, not across species (Fig. S5d, e), indicating that our data is equally reliable for small and large regions.

**Fig. 5:**
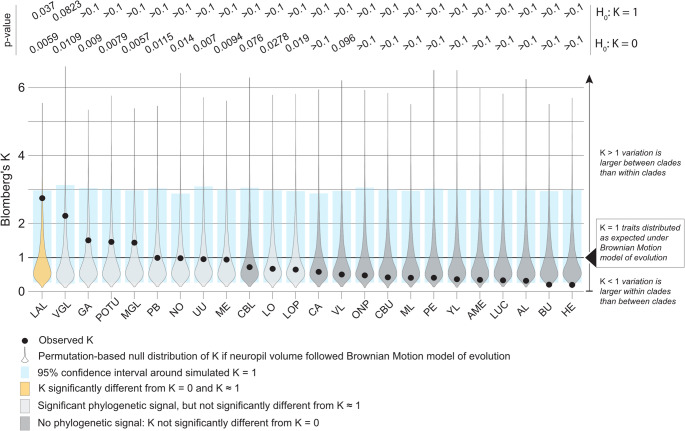
Phylogenetic signal for all defined neuropils, calculated as Blomberg’s *K*. $K = 1$ indicates that neuropil volumes are distributed as expected under a Brownian Motion (BM) model of evolution, while $K < 1$ indicates that closely related species are less similar than expected under a Brownian motion model, and $K > 1$ indicates that they are more similar than expected. Significance values are given for hypothesis tests of $K = 0$ (no phylogenetic signal) and $K = 1$ (*K* follows BM model of evolution)

To understand how brains adapt to different ecologies, it is imperative to first understand how they are expected to evolve if there were no such adaptations, i.e. to quantify the impact of phylogeny. We therefore estimated the phylogenetic signal using Blomberg’s *K*, and compared the observed *K* to a permutation-based null-distribution of *K* simulated under a Brownian motion model of evolution (BM). We complemented this by testing the observed *K* against randomly shuffled values across the phylogeny, to estimate whether phylogeny has a significant impact on neuropil volume. We find that 11 out of 24 neuropils have an observed *K* that is significantly different from 0, indicating that phylogeny has a significant impact on neuropil volume (Fig. 5). Of those 11 neuropils, 10 are distributed as expected under a BM model of evolution ($K \approx 1$), while only the LAL is significantly different from $K \approx 1$. This indicates that the LAL is the only neuropil that does not evolve as expected under a BM model of evolution, and may be more strongly affected by phylogeny or environmental pressures. More generally, the finding that almost half of all neuropils included in this study are significantly impacted by phylogeny ($K \ne 0$) highlights the need to correct all following analyses for phylogenetic distance.

### A set of evolutionarily stable core neuropils

**Fig. 6:**
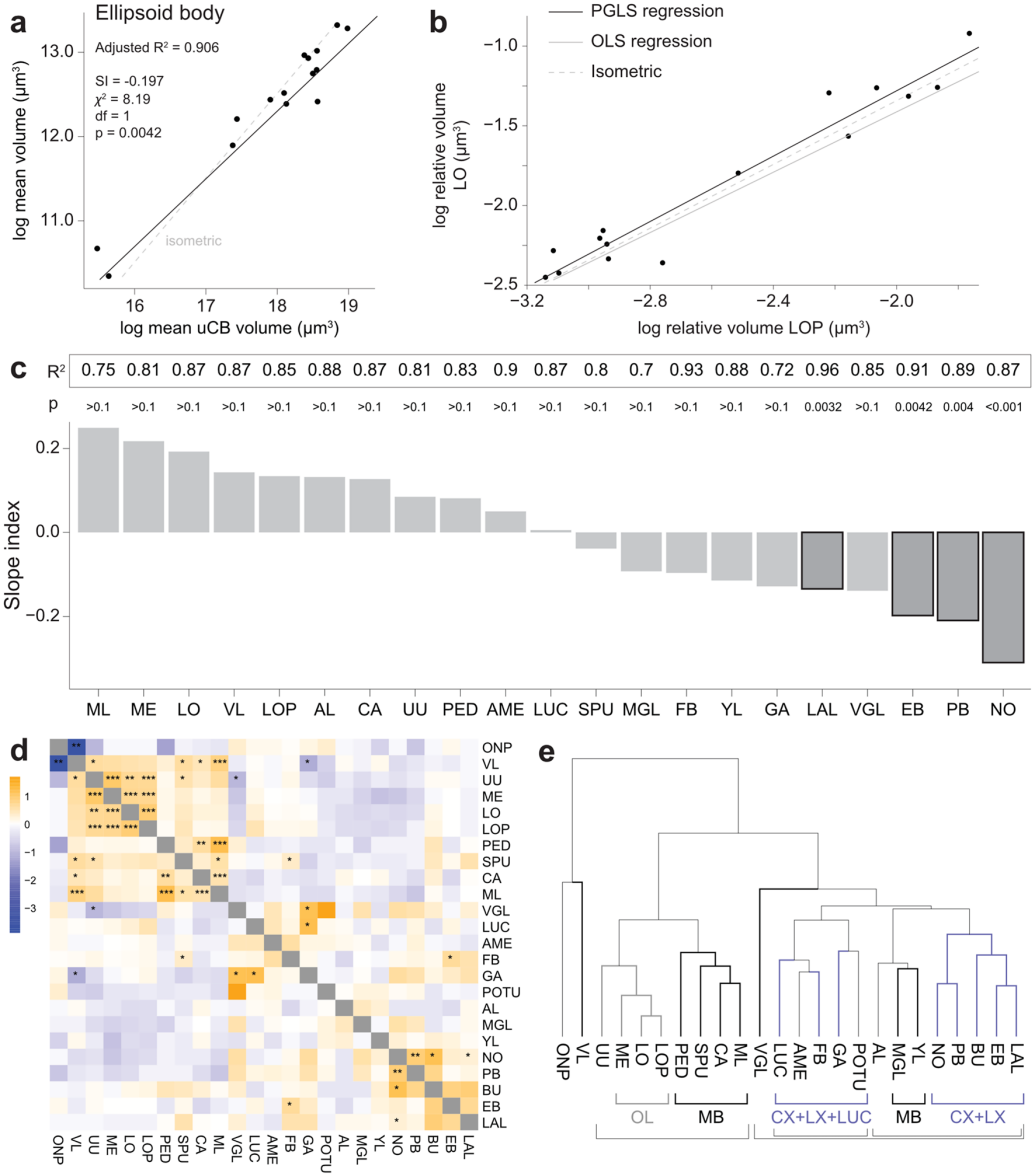
Scaling analysis. **a** PGLS model of the ellipsoid body compared to isometric scaling (where $$b = 1$$) reveals a significantly shallower slope *b*. **b** Comparison of PGLS and OLS regressions, for relative volumes lobula (LO) vs. lobula plate (LOP). OLS follows the data more closely, while PGLS accounts for phylogenetic distance. Neither model is significantly different from isometry, indicating that LO and LOP scale in synchrony. **c** Slope index for all neuropils with an adjusted PGLS $$R^2 > 0.6$$ compared to isometry. Outlined bars indicate significance based on the Chi-squared test. **d** Correlation matrix of slopes, calculated using PGLS as in *b*. **e** Dendrogram based on hierarchical clustering of ***d*** reveals clustering according to superregions. $$* = 0.05> p > 0.01$$, $$** = 0.01> p > 0.001$$, $$*** = p < 0.001$$, n.s. = not significant

To examine how neuropil volumes change across species, we first analysed how they scale compared to an isometric expectation and with respect to each other, i.e. we computed slope indices for each neuropil across the data set (Fig. 6). Note that in all following analyses of the MB lobes, we could not include the Monarch butterfly, as its lobes are fused together, making identification of individual lobes highly uncertain based on synaptic labeling alone.

When comparing slopes to isometric scaling (example in Fig. 6), we found that no neuropil scales significantly more steeply than isometric, but several neuropils scale less steeply (Fig. 6c). Among these are three of the four CX neuropils (ellipsoid body, protocerebral bridge, and noduli), as well as the lateral accessory lobe of the LX. We conclude that these regions have a conserved function that cannot be augmented by adding more neural tissue, thus yielding limited gains from increasing their size beyond what is needed to achieve their essential functional roles.

Beyond comparing slope indices to isometric scaling, we also performed pairwise slope comparisons. We reasoned that if different neuropils scale similarly across the lepidopteran phylogenetic tree, this could suggest that these regions evolve in conjunction and might thus form functional units, possibly distinct from the anatomical units they belong to. To test this, we used PGLS regression for all neuropils against each other (example in Fig. 6b), transformed the resulting slope estimates into a distance matrix, then performed hierarchical clustering. The resulting correlation matrix and dendrogram (Fig. 6d, e) provide an insight into neuropils that scale in conjunction across lepidopteran evolution, and might therefore be thought of as potential functional units. Overall, we find a mix of positive and negative correlations that yield clusters of neuropils. While the positively linked clusters broadly recapitulate anatomical superregions as defined by Ito et al (2014), there are some interesting exceptions. For example, the upper unit of the AOTU forms a tight cluster with the large neuropils of the OL, while the lower unit complex of the AOTU is more closely associated with the neuropils of the CX. Similarly, the accessory medulla, while anatomically part of the OL, does not cluster with the remaining OL neuropils, supporting its distinct functional role in housing the central circadian clock of the insect brain.

Surprisingly, the MB did not form one contiguous cluster, but was split into two main parts. One sub-cluster was formed by the calyx, pedunculus, spur and main medial lobes. This MB cluster was more closely associated with the visual neuropils. The second MB cluster consisted of the medial and vertical $\gamma$-lobes and the Y-lobe, which associated with the CX and LX neuropils as well as the AL. In combination, these findings support a pronounced multimodality of the MB in lepidopterans.

We also find significant negative correlations, where increased investment in one neuropil is linked to a decrease in investment in the other. These are found for the vertical lobe components of the MB, which negatively correlate with the gall and the upper division of the AOTU.

In summary, the slope index analysis indicates that the CX and LX scale significantly less steeply than expected from isometry. This suggests that after these regions have reached a specific relative size, further size increase results in limited functional gains, consistent with a role as evolutionarily stable, core regions of the insect brain. These findings are similar to data from *Heliconius* butterflies, in which the CX and AOTU were evolutionary stable across differences in ecologies, despite the MB showing accelerated rates of evolution in the same species (Farnworth et al. 2025).

### Diurnal activity period is reflected by distinct neuropil investment patterns

**Fig. 7:**
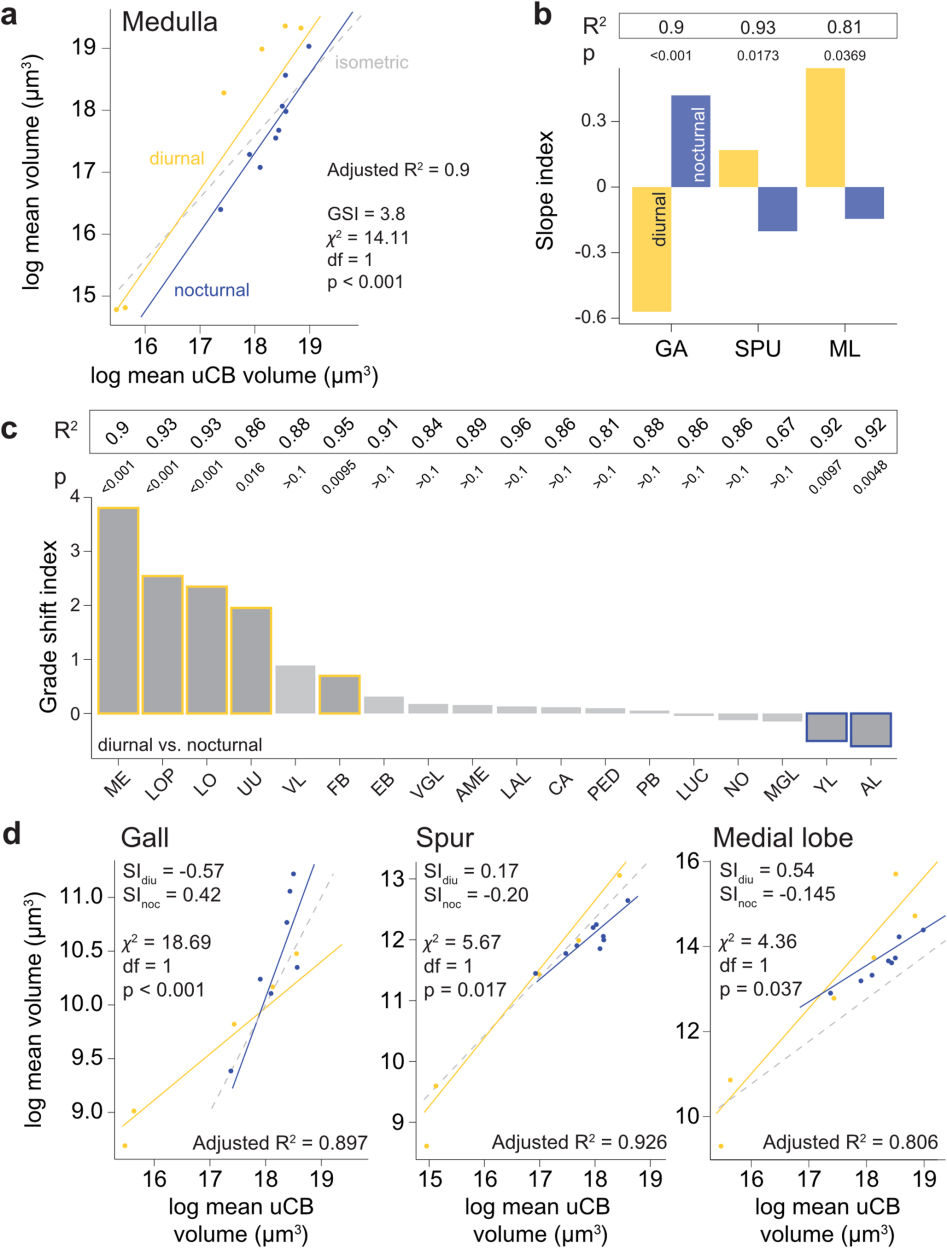
Impact of activity period on neuropil volumes. **a** PGLS model for the medulla of the optic lobe shows a significant shift of the y-intercept (grade shift) towards systematically larger volumes in diurnal Lepidoptera. **b** Slope index for neuropils which had significantly different slopes *b*. **c** Grade shift index for all neuropils with a PGLS $$R^2 > 0.6$$. Yellow/blue outlines indicate significant grade shifts. **d.** PGLS models for the lower unit complex of the AOTU and gall of the LX showing significantly different slopes *b*. Yellow = diurnal, blue = nocturnal. Significance based on the Chi-squared test. $$* = 0.05> p > 0.01$$, $$** = 0.01> p > 0.001$$, $$*** = p < 0.001$$, n.s. = not significant

To understand how a species’ ecology influences neuropil volume, we began by considering activity period, which is known to impact the relative volumes of primary sensory neuropils (e.g. Keesey et al. 2019; Stöckl et al. 2016). To evaluate how brain regions scale across different species and whether they scale differently depending on whether a species is nocturnal or diurnal, we computed a phylogenetically corrected grade shift index (gsi). This measure captures the difference in y-intercept between two phylogenetically corrected regressions (Fig. 7a), indicating whether neuropils are systematically larger in diurnal (gsi >0) than in nocturnal moths (Fig. 7c).

As this method did not yield a significant regression for the bulb, this region could not be further analysed. Additionally, the grade shift index is only valid if the slopes of the regressions are not significantly different. However, we did find significantly different slopes in three neuropils (Fig. 7b): The gall of the LX ($\chi ^2$ = 18.69, df = 1, p < 0.0001), the spur of the MB ($\chi ^2$ = 5.67, df = 1, p = 0.017), and the medial lobe of the MB ($\chi ^2$ = 4.36, df = 1, p = 0.037; Fig. 7d). The spur and medial lobe scale significantly more steeply in diurnal moths than in their nocturnal relatives, and vice versa for the gall.

The majority of neuropils passed the conditions for the grade shift analysis. For those, we found that the medulla, lobula, lobula plate, and upper unit of the AOTU are significantly larger in diurnal moths, while the antennal lobe is significantly larger in nocturnal moths (Fig. 7c, S2). While these differences in sensory neuropils were expected from previous studies (Stöckl et al. 2016; Keesey et al. 2019), we also find surprising differences in two integrative neuropils: the fan-shaped body ($\chi ^2$ = 6.74, df = 1, p = 0.0095) and the Y-lobe ($\chi ^2$ = 6.697, df = 1, p = 0.0097). While the fan-shaped body was larger in diurnal species, the Y-lobe was larger in nocturnal species.

### The footprint of migratory ability on brain anatomy

**Fig. 8:**
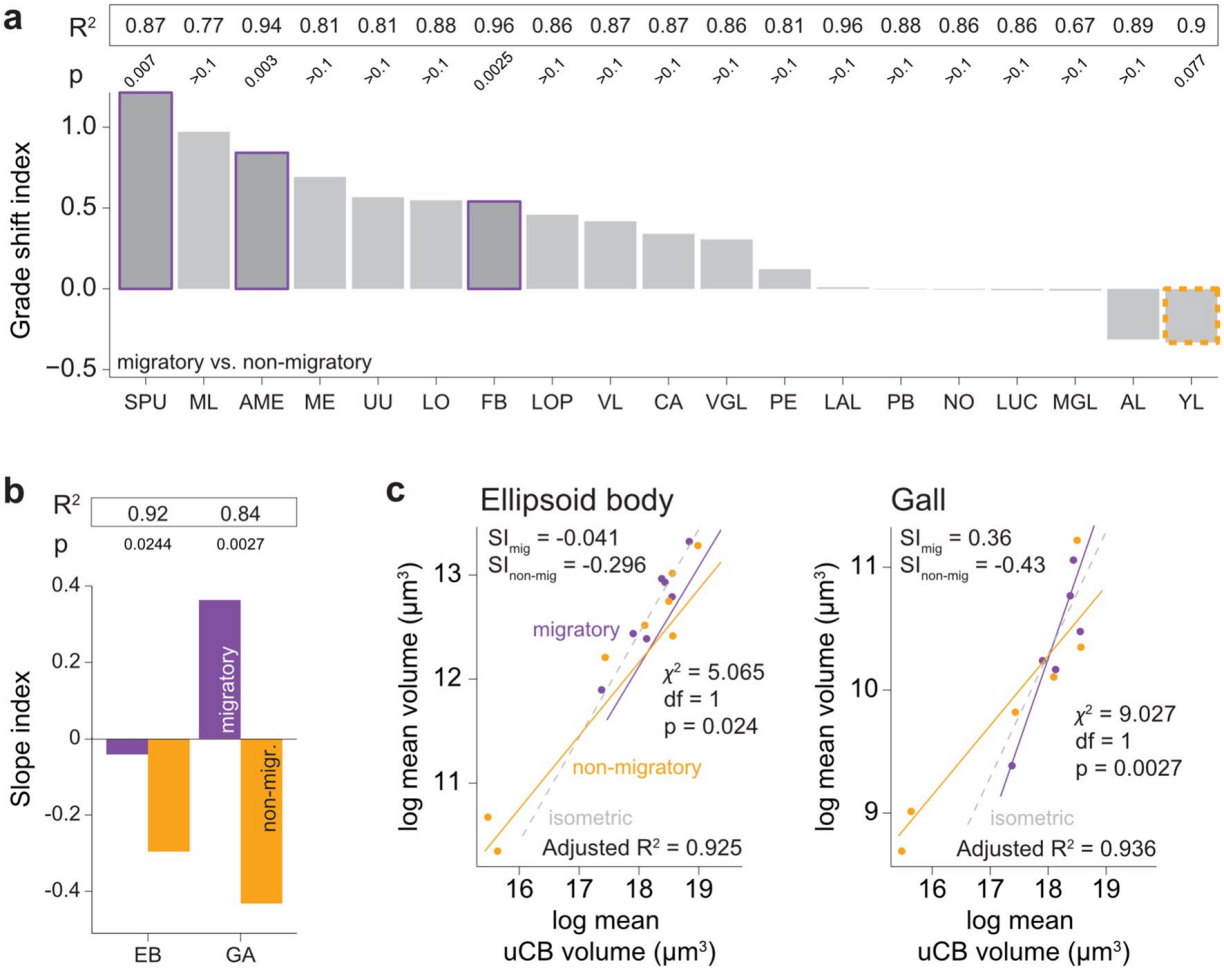
Impact of migratory ability on neuropil volumes. **a** Grade shift index for all neuropils with a PGLS $$R^2 > 0.6$$. Purple/orange outlines indicate significant grade shifts. **b** Slope index for neuropils which had significantly different slopes *b*. **c** PGLS models for the ellipsoid body of the CX and gall of the LX showing significantly different slopes *b*. Orange = migratory, purple = non-migratory. Significance based on the Chi-squared test. $$* = 0.05> p > 0.01$$, $$** = 0.01> p > 0.001$$, n.s. = not significant

After identifying the expected changes in the size of sensory brain regions depending on activity period, we asked if more complex behavioral traits are also reflected in volumetric changes of brain regions. We therefore performed an analogous analysis for migratory as compared to non-migratory Lepidoptera (Fig. 8a).

Interestingly, the gall again has significantly different slopes (Fig. 8b, c), along with the ellipsoid body, both being steeper in migrants (EB: $\chi ^2$ = 5.065, df = 1, p = 0.024; GA: $\chi ^2$ = 9.027, df = 1, p = 0.0027).

All other neuropils were subjected to grade shift analysis. While the majority of neuropils do not show strong differences between migratory versus non-migratory species, we found significant grade shifts in the MB spur ($\chi ^2$ = 7.299, df = 1, p = 0.0069), the accessory medulla ($\chi ^2$ = 8.81, df = 1, p = 0.003), and the fan-shaped body ($\chi ^2$ = 9.16, df = 1, p = 0.0025). All three neuropils are systematically larger in migratory than non-migratory moths. Interestingly, the Y-lobe’s gsi had a non-significant tendency to be enlarged in non-migratory species ($\chi ^2$ = 3.137, df = 1, p = 0.077).

To double-check that the differences found for the slope indices were not due to an oversized influence of the two smallest moths, *E. semipurpurella* and *L. capitella*, we performed a leverage analysis, where the PGLS residuals were normalised to the sum of all residuals. Indeed, no datapoint fell above the cut-off of the mean plus two standard deviations for the EB, while for the GA, only *S. ocellatus* had slightly higher than expected leverage (data not shown). We also checked PGLS models that included both diurnal/nocturnal as well as migratory/non-migratory groups and found that in these models, neither group had a significant effect on the slope or intercept of the regressions, and there were no significant interactions. We therefore conclude that the slope differences in the GA in migratory and non-migratory moths are not confounded by the slope differences in the same neuropil in day- versus night-active moths.

## Discussion

In this study, we present a qualitative and quantitative comparison of the brains of 15 lepidopteran species, which were chosen to represent a variety of clades along the phylogenetic tree. As an outgroup species, we included the caddisfly *R. nubila*. To our knowledge, the resulting brain model is the first full account of a trichopteran brain to date, revealing many similarities to lepidopteran brains. Yet, a set of anatomical features emerged that was exclusively shared across all analysed lepidopteran species, but was distinct in Trichoptera. These common brain characteristics appear to define the ground plan of the lepidopteran brain.

We further examined neuropil volumes in a phylogenetic context, and found several neuropil groups that stand out across different analyses. As the function of some of these neuropils is as yet unknown, our analysis provides a first indication of functional pathways they may belong to, opening up access points for future studies.

### Qualitative neuropil variations across the Lepidoptera

Arguably the most dramatic qualitative difference among the studied brains was the presence of a large, distinct optic glomerular complex in the Monarch butterfly. While this region was unique to the Monarch across the species covered by our study, a small spherical region found near the ventral tip of the lobula in *V. cardui* occupied a similar area in the brain of that species. It was reminiscent of the ventral lobula of *Heliconius* (Montgomery et al. 2016) and *Papilio xuthus* (Kinoshita et al. 2015), which was suggested as a homologue of the Monarch butterfly optic glomerular complex. Anatomically similar regions of variable size were also recently identified in a group of ithomiine butterflies (Wainwright et al. 2023).

As butterflies are the most visual of all lepidopteran insects, a role of these neuropils specific for the ecology of a visual pollinator seems likely. Indeed, in the Swallowtail *Papilio xuthus* and in *Heliconius* butterflies, the ventral lobula was found to contain visual projection fibers directly linking the medulla to the visual regions of the MB calyx (Kinoshita et al. 2015; Couto et al. 2023). This suggests a dedicated pathway used to deliver information for visual learning in butterflies that involves the ventral lobula and the possibly homologous optic glomerular complex. This is notably distinct from the neuropils described as optic glomeruli in flies, which serve as output regions of columnar projection neurons from the lobula (Strausfeld and Okamura 2007) and are not interconnected with the MB.

Interestingly, dye injections into the ventral lobula of ithomiine butterflies revealed no link to the MB, but rather showed projections to the bulb of the LX (Wainwright et al. 2023). This fundamentally different projection pattern indicates a function in the context of compass navigation in those species, suggesting that ventral-lobula-like regions might not all be homologous and could have evolved and been lost several times within butterflies. The interface of the optic lobe and central brain thus emerges as a possible hotspot for evolutionary change, possibly driven by diverse ecological needs.

Another set of visual regions also showed qualitative differences across the examined species: the AOTU and the posterior optic tubercle (POTU). As shown for species from other insect orders, the POTU is sporadically present and absent without obvious phylogenetic trend. For instance, it is missing in the fruit fly *Drosophila* (Ito et al. 2014; Hulse et al. 2021) and two species of dung beetles (el Jundi et al. 2018), but is present in bees. Its presence in many hemimetabolous insects (Homberg et al. 1991) indicates that this region is ancestral and might have been repeatedly lost in holometabolous species. Lepidoptera reflect this general pattern and appear to have lost, or significantly reduced the POTU in some species, specifically in hawkmoths and, possibly, in early-diverging Lepidoptera. It remains to be seen whether this neuropil is truly absent in these insects, or whether it has been absorbed into surrounding neuropils. Examining the morphology of $\Delta$7 neurons (also called TB1 neurons) of the protocerebral bridge would resolve this question. While these cells send sidebranches into the POTU if it exists (e.g. Heinze and Homberg 2007), they lack those branches in species without this region (e.g. flies and beetles)(el Jundi et al. 2018; Wolff et al. 2015). Anatomically, a direct link between the POTU and the accessory medulla was shown in locusts (Homberg and Würden 1997; el Jundi and Homberg 2010), suggesting that circadian information travels along this pathway towards the CX head direction circuit. A prominent POTU could thus indicate an important role for circadian information in the context of navigation and orientation, e.g. to time compensate flight headings during long distance migration.

The observed differences in the AOTU were restricted to the lower unit complex, which varied in number, shape and arrangement of subunits. This was in contrast to the conserved upper unit of the AOTU, which co-varied in volume with the optic lobe, but which was consistently present as a single neuropil. While the upper unit has been shown to be involved in the processing of colour information in bees (Mota et al. 2013), the lower unit complex is the main relay station for visual, orientation-relevant directional information (Hulse et al. 2021; Heinze and Reppert 2011; Homberg and el Jundi 2014). Various distinct sources of visual navigation cues are relayed via parallel pathways, each passing through one subunit of the lower unit complex of the AOTU. Allocating different amounts of neural tissue to any such pathway would suggest an increased relevance of the relayed type of information for the orientation ability of a species. Similarly, insulating pathways from each other by segregating subunits might reduce interference between them to minimise noise and allow for targeted gain regulation via neuromodulators. The organization of the lower unit complex might thus directly reflect the relative importance of different types of spatial information for a species.

As the AOTU lower unit complex undergoes prominent qualitative changes, it is clearly a region with unusually high evolvability. Such an evolutionary hotspot could be a promising model for investigating how differences in ecological characteristics affect neural processing.

### Impact of ecological traits on neuropil volumes

While a handful of evolutionary innovations yield qualitative differences across Lepidoptera, the majority of changes were of a quantitative nature, resulting in volumetric differences. Those differences recapitulated phylogenetic relationships to a large extent, but more interestingly, also reflected ecological characteristics.

We investigated the impact of two ecological adaptations on neuropil volume: activity period (diurnal or nocturnal), and migratory behaviour, expecting that the former would be primarily reflected in changes in the primary sensory neuropils. Previous studies demonstrated that day-active species, which rely primarily on vision, have relatively larger visual neuropils, whereas nocturnal species have relatively larger antennal lobes, reflecting their higher reliance on olfaction (Stöckl et al. 2016; Rivas-Sánchez et al. 2024; Keesey et al. 2019). We confirm these findings in our dataset, where the medulla, lobula and lobula plate, as well as the upper unit of the AOTU, are larger in diurnal Lepidoptera. Interestingly, we also found that the fan-shaped body is significantly larger in diurnal species, while the Y-lobe of the MB is significantly larger in nocturnal moths. The Y-lobe in particular is a lepidopteran innovation of unknown function that does not exist in their closest sister clade, the Trichoptera.

In contrast to activity period, we expected migratory behaviour to be reflected in integrative neuropils of the central brain. Although a previous study failed to find significant volumetric changes in neuropils of the compass pathway (AOTU, CX and LX) when comparing two closely related species of noctuid moths (de Vries et al. 2017), our broader phylogenetic analysis revealed three neuropils that were significantly and systematically enlarged in migratory species: the fan-shaped body of the CX, the accessory medulla of the OL, and the spur of the MB. The fan-shaped body is the least surprising of the three, as it is known to process orientation and navigation signals to compute navigational goals (Mussells-Pires et al. 2024). Generally, the circuits of the fan-shaped body have been proposed to encode multiple parallel goal directions, each of which is gated onto steering cells by a specific set of modulatory inputs (Heinze 2023; Hulse et al. 2021; Matheson et al. 2022). One such goal could be a genetically encoded vector that guides insects during their migration (Honkanen et al. 2019). It is conceivable that migratory insect species need to invest more in the fan-shaped body to enable the encoding of genetically fixed navigational goals, adding to the circuits needed to drive local foraging behaviors when the insects are in a non-migratory state.

The accessory medulla houses the circadian clock, as shown for cockroaches and flies (Reischig and Stengl 2003; Reinhard et al. 2024). Based on the anatomical and molecular similarity between insect species (Numata et al. 2015), this region is most likely generally involved in circadian control. If all migrating Lepidoptera possess a time-compensated celestial compass, as shown for the Monarch butterfly (Reppert et al. 2016; Froy et al. 2003; Mouritsen and Frost 2002), this region can be expected to play a vital role in keeping those insects on track during their migrations. Investing more neural resources in circadian control circuits might improve the reliability of the circadian clock or add modulatory functions required for seasonal migrations that go beyond the needs of non-migratory species.

While grade shifts show consistent changes in investment into neuropil volume, slope differences are harder to interpret. However, we found several neuropils for which the slope of the allometric regression significantly differed across ecologies, indicating fundamental differences in scaling and thus patterns of neural investment. This was the case for the CX ellipsoid body, the MB spur and medial lobe, and the LX gall. Interestingly, the gall scaled significantly more steeply in both nocturnal and migratory species. This neuropil is an integral, yet not well understood, part of the head direction network in the CX. It receives axons from ellipsoid body head direction cells (EPG neurons) and provides the basis for a feedback loop between specific ring neurons (ER neurons) and the EPG cell dendrites in the ellipsoid body (Hulse et al. 2021). While the function of this loop is unknown, such feedback could have a stabilizing effect on a head direction bump that has to be sustained even if sensory input provided by the ER neurons is noisy (Hulse et al. 2021; Heinze and Reppert 2011; Heinze 2024). Our finding that the gall scales more steeply in nocturnal and migratory Lepidoptera may reflect differences in how the demands on these circuits scale with animal size to maintain precise orientation and navigation in dim light conditions and over vast distances.

### Hotspots of evolutionary change in the lepidopteran brain

Across our analyses, several neuropils stood out as more variable and flexible. These include the lower unit complex of the AOTU, the POTU, the optic glomerular complex/ventral lobula, the Y-lobes and spur of the MB, the fan-shaped body of the CX, the gall of the LX and the accessory medulla. While functional data exist for most of these regions as discussed above, the role of the Y-lobes and spur are unclear.

We have shown that the Y-lobe is enlarged in nocturnal Lepidoptera, but tends to be smaller (albeit not significantly, p = 0.077) in migratory species. Our clustering analysis also showed that, together with the medial gamma lobe of the MB, the Y-lobe is linked most closely with the antennal lobes, clustering distinctly from the remaining parts of the MB. A similar separation was also shown in *Spodoptera littoralis*, a noctuid moth, in which the Y-lobe is supplied by class III Kenyon cells that are distinct in shape from the Kenyon cells that supply the main lobes of the MB (Sjöholm et al. 2005). In that species, their dendrites cover a wide, central area of the MB calyx, without clear correspondence to input regions associated with specific sensory modalities. In contrast, our data indicate that the Y-lobes are more tightly linked to olfaction than to other modalities, in particular vision, suggesting a potentially specific role in olfactory learning. As for early stage olfactory processing, olfactory learning thus appears more closely associated with nocturnal ecologies. Additionally, our data suggest that migratory ability correlates with a reduced requirement for olfactory learning, which is supported by findings showing that long-distance migrations of insects are largely visually guided.

The spur’s function is as yet unknown, but data from *Spodoptera* shows that it comprises a third arborization domain of the Kenyon cell axons that make up the vertical and medial domains of the three main MB lobe systems ($\alpha /\beta$, $\alpha '/\beta '$, and $\gamma$) (Sjöholm et al. 2005). In *Drosophila*, the MB lobes are divided into 15 functional compartments that are defined by the projection fields of the principal MB output neurons (MBONs) and overlapping dopaminergic reward neurons (DANs) (Li et al. 2020). We therefore propose that a dedicated set of MBONs and DANs connect to the Kenyon cells in the lepidopteran spur, driving a specific set of learning processes that might be more relevant to migratory and diurnal ecologies. Investigating the nature of the associated MBONs and DANs in the spur might thus be a promising starting point to illuminate long-term memory processes relevant to migratory behavior.

In general, the association of individual MB lobes with ecological traits as well as the lack of consistent volumetric linkage between MB components suggests mosaic evolution of the lepidopteran MB. This finding is supported by similar data from *Heliconius* butterflies, in which the MB also shows selected expansion of individual lobes, changes that were linked to expansion of the underlying Kenyon cell populations (Farnworth et al. 2024).

Interestingly, the majority of highly variable neuropils are small neuropils that act as relays to larger, more conserved, brain regions: The gall mediates recurrent feedback for CX neurons, the POTU passes internal clock information from the accessory medulla to CX circuits, and the lower unit complex is a well-known relay for orientation-related visual cues that pass from OL output neurons to the CX. We suggest that such highly adaptable hotspots may enable evolution to modulate the bandwidth of critical pathways towards integrative brain regions such as the CX, thus modifying functionality without having to alter these important processing centres directly. Similarly, the spur of the MB might house a dedicated set of reward and output neurons suited to form an associative pathway independent of the default set of MB learning circuits. Such an additional circuit could easily evolve to adapt to specific needs, without disrupting established functions of the main MB.

### Broader insights by taking a phylogenetic perspective

Although a wide range of species has traditionally been used as basis for comparative insect neuroanatomy (e.g. Hanström 1926; Holmgren 1916; Flögel 1878), recent studies linking ecological differences to brain size using modern methods have focused on closely related species, or even different behavioral stages within the same species. The majority of these studies also concentrated on a limited set of neuropils within the brain. Our results show that taking a broader phylogenetic view and increasing the resolution of brain reconstructions allows us to tease apart phylogenetic and ecological effects across the entire brain. Consequently, our anatomical data enable us to pinpoint evolutionarily conserved versus flexible neuropil groups, and even suggest initial functional hypotheses for neuropils that have not been functionally investigated. This analysis also allows us to gain valuable insights into general mechanisms of brain evolution, such as revealing "hotspots" for evolutionary change. Such hotspots could serve as valuable starting points for focused circuit mapping using comparative connectomics, as well as for functional circuit analysis in non-standard model species. We therefore believe that taking a phylogenetic view is a powerful and informative approach that can significantly add to our understanding of brain anatomy and function.

## Supplementary Information

Below is the link to the electronic supplementary material.

Supplementary file 1 (pdf 12342 KB)

## Data Availability

Anatomical data, including 3D-reconstructions and confocal image stacks, can be found on the insect brain database: http://www.insectbraindb.org. All code and quantitative data are freely available on GitHub, https://github.com/stanleyheinze/Lepidopteran_brain_evolution

## Abbreviations

R.nub: *Rhyacophila nubila*
E.sem: *Eriocrania semipurpurella*
A.atr: *Abantiades atripalpis*
L.cap: *Lampronia capitella*
D.ple: *Danaus plexippus*
B.any: *Bicyclus anynana*
V.car: *Vanessa cardui*
S.oce: *Smerinthus ocellatus*
M.sex: *Manduca sexta*
M.ste: *Macroglossum stellatarum*
D.elp: *Deilephila elpenor*
A.gam: *Autographa gamma*
A.inf: *Agrotis infusa*
N.pro: *Noctua pronuba*
M.pal: *Mythimna pallens*
O.got: *Orthosia gothica*
AL: Antennal lobe
AME: Accessory medulla
AOTU: Anterior optic tubercle
BU: Bulb
CA: Calyx
CX: Central complex
EB: Ellipsoid body
FB: Fan-shaped body
GA: Gall
GNG: Gnathal ganglion
LA: Lamina
LAL: Lateral accessory lobe
LO: Lobula
LOP: Lobula plate
LU: Lower unit
LUC: Lower unit complex
LX: Lateral complex
MB: Mushroom body
ME: Medulla
MGL: Medial $\gamma$ lobe
ML: Medial lobe
MU: Medial unit
NO: Nodulus
NU: Nodular unit
OCN: Ocellar neuropil
OGC: Optic glomerular complex
OL: Optic lobe
PB: Protocerebral bridge
PED: Peduncle
POTU: Posterior optic tubercle
SP: Strap
SPU: Spur
uCB: Undefined central brain
UU: Upper unit
VGL: Vertical $\gamma$ lobe
VL: Vertical lobe
YL: Y-lobe
BM: Brownian motion
GSI: Grade-shift index
OLS: Ordinary least squares
PGLS: Phylogenetic generalised least squares
SI: Slope index
