# Supplementary material for: The evolution of lepidopteran brain morphology
Source: J Comp Physiol A Neuroethol Sens Neural Behav Physiol. 2025 Dec 22;212(2):409–32. doi: 10.1007/s00359-025-01787-w (PMC13086899; doi:10.1007/s00359-025-01787-w)
Supplement: Supplementary file 1 — (pdf 12342 KB) [file 359_2025_1787_MOESM1_ESM.pdf]

Lepidopteran brain evolution

Supplementary Figures

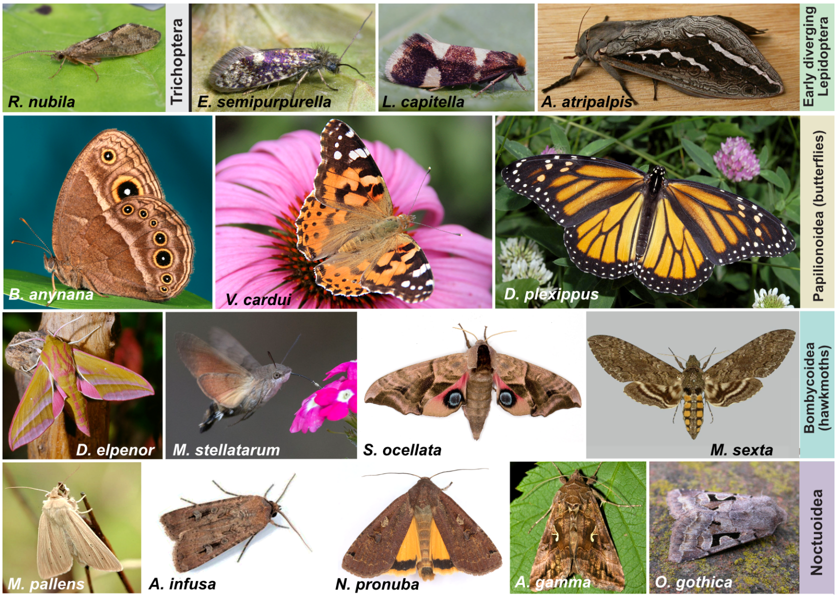

Fig. S1 Photographs of each species used in this study. Sources: *R. nubila*: Hallvard Elven, Naturhistorisk museum, Universitetet i Oslo, CC BY 4.0, via Wikimedia Commons; *E. semipurpurella*: Patrick Clement from West Midlands, England, CC BY 2.0, via Wikimedia Commons ; *L. capitella*: Cossus, CC BY-NC 4.0, via iNaturalist; *A. atripalpis*: dhobern, CC BY 2.0, via Wikimedia Commons; *B. anynana*: William Piel, source; *V. cardui*: Jean-Pol Grandmont, CC BY 3.0; *D. plexippus*: Kenneth Dwain Harrelson, CC BY-SA 3.0; *D. elpenor*: Jean Pierre Hamon, CC BY-SA 3.0; *M. stellatarum*: Jerzy Strzelecki, CC BY-SA 3.0; *S. ocellata*: Jerzy Strzelecki, CC BY-SA 4.0, via Wikimedia Commons; *M. sexta*: The Trustees of the Natural History Museum, London, CC BY 3.0, via Wikimedia Commons; *M. pallens*: James K. Lindsey, CC BY-SA 2.5, via Wikimedia Commons; *A. infusa*: Ajay Narendra, with permission; *N. pronuba*: Janet Graham, CC BY 4.0, via Wikimedia Commons; *A. gamma*: Olei, CC BY-SA 2.5, via Wikimedia Commons; *O. gothica*: Donald Hobern, CC BY 2.0, via Wikimedia Commons.

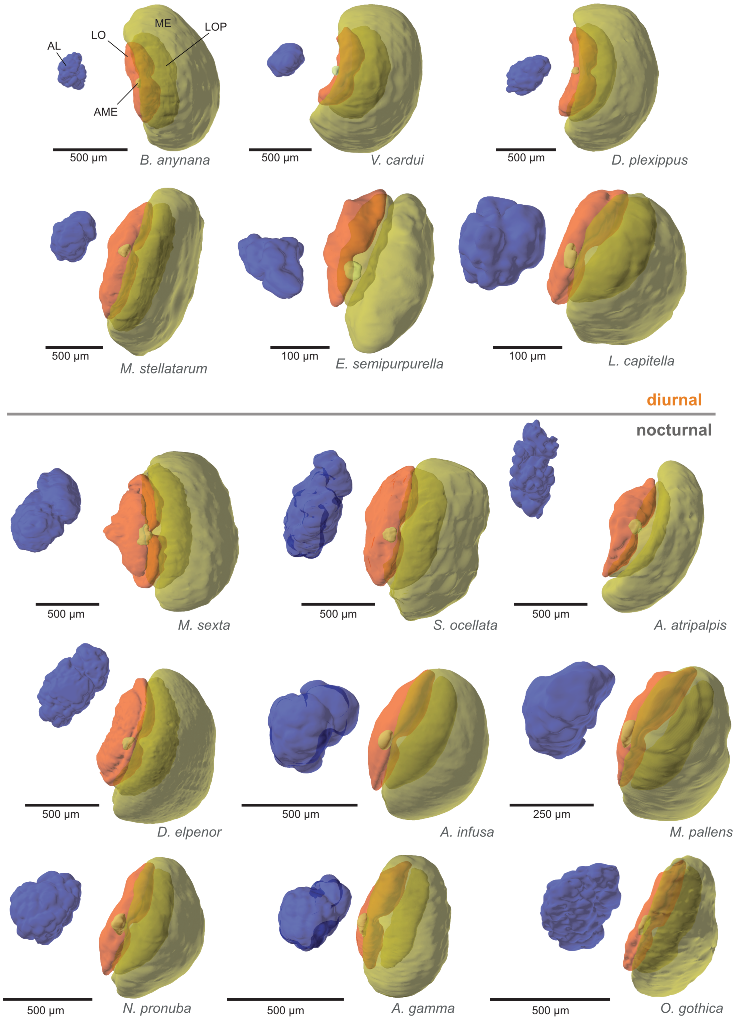

Fig. S2 3D reconstructions of each species' optic lobe (OL) and antennal lobe (AL). Note the differences in relative size between AL and OL for nocturnal and diurnal species. ME, medulla; LO, lobula, LOP, lobula plate, AME, accessory medulla. Images generated with insectbraindb.org

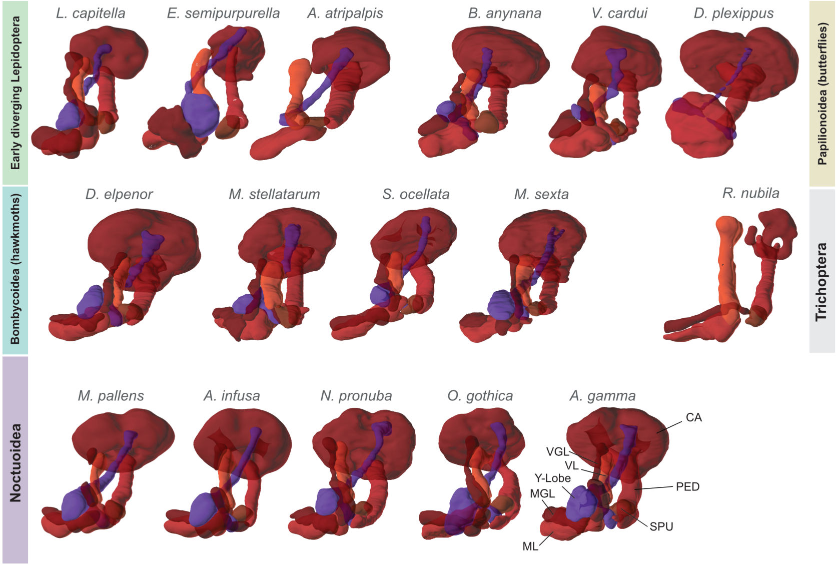

Fig. S3 3D reconstructions of the mushroom bodies of each species used in this study. All samples are scaled to matching display sizes. CA, calyx; PED, pedunculus; ML, medial lobe; VL, vertical lobe; MGL, mdial gamma lobe; VGL, vertical gamma lobe; SPU, spur. Images generated with insectbraindb.org

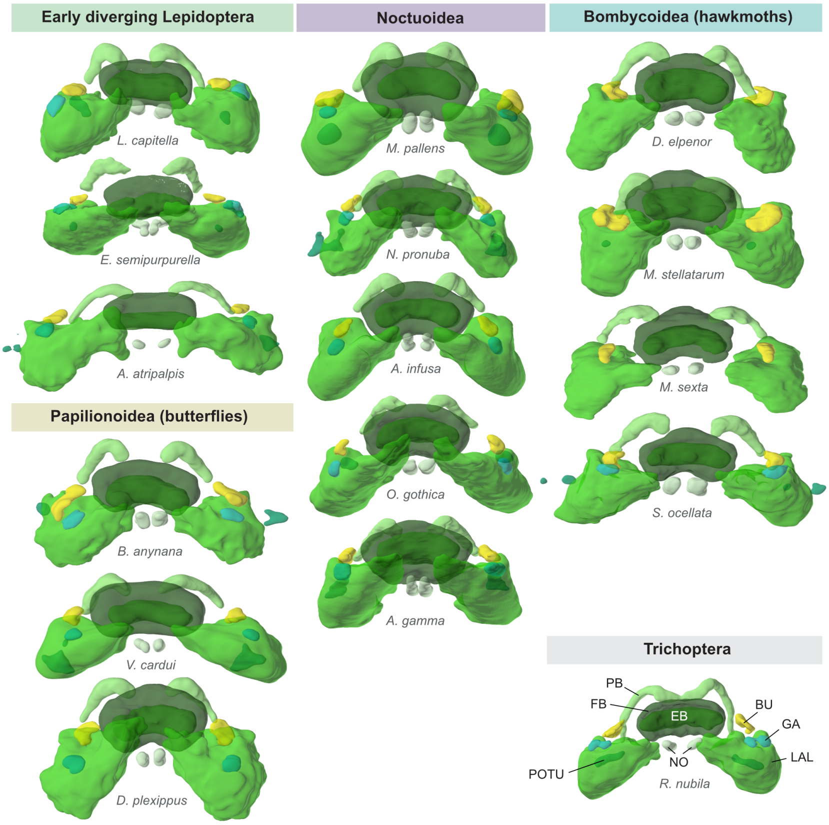

Fig. S4 3D reconstructions of the central and lateral complexes (CX and LX) of each species used in this study. All samples are scaled so that the width of the ellipsoid body (EB) matches between species. FB, fan-shaped body; EB, ellipsoid body; PB, protocerebral bridge; NO, noduli; BU, bulb; GA, gall; LAL, lateral accessory lobe; POTU, posterior optic tubercle. Images generated with insectbraindb.org

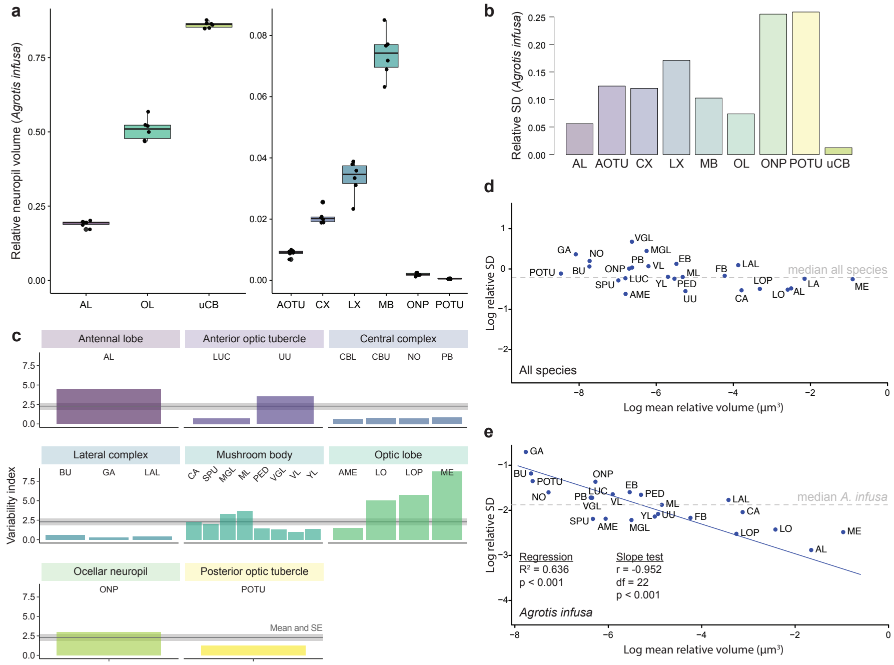

Fig. S5 Variability of brain region volumes across and within species. a. Relative volume of all superregions across species, divided into large and small neuropils. **b.** Relative standard deviation of superregions within *Agrotis infusa*, calculated as the SD divided by the mean. **c.** Variability index for all neuropils, calculated as the relative inter-specific SD divided by the relative SD within *A. infusa* -1. **d.** Log relative SD across species plotted against the log mean relative volume. Broken grey line indicates median log relative SD across species. **e.** Log relative SD within six samples of *A. infusa*, plotted against the log mean relative volume. Broken grey line indicates median log relative SD within *A. infusa*. Note that smaller neuropils tend to have a larger relative SD within *A. infusa*, but the same is not true across species, as shown in d.
